# Chromosome-specific NOR inactivation explains selective rRNA gene silencing and dosage control in *Arabidopsis*

**DOI:** 10.1101/gad.273755.115

**Published:** 2016-01-15

**Authors:** Chinmayi Chandrasekhara, Gireesha Mohannath, Todd Blevins, Frederic Pontvianne, Craig S. Pikaard

**Affiliations:** 1 Department of Molecular and Cellular Biochemistry, Indiana University, Bloomington, Indiana 47405, USA;; 2 Department of Biology, Indiana University, Bloomington, Indiana 47405, USA;; 3 Howard Hughes Medical Institute, Indiana University, Bloomington, Indiana 47405, USA

**Keywords:** transcription, rRNA gene subtypes, nucleolus organizer region, gene silencing, chromatin

## Abstract

In eukaryotes, scores of excess ribosomal RNA (rRNA) genes are silenced by repressive chromatin modifications. Given the near sequence identity of rRNA genes within a species, it is unclear how specific rRNA genes are reproducibly chosen for silencing. Using *Arabidopsis thaliana* ecotype (strain) Col-0, a systematic search identified sequence polymorphisms that differ between active and developmentally silenced rRNA gene subtypes. Recombinant inbred mapping populations derived from three different ecotype crosses were then used to map the chromosomal locations of silenced and active RNA gene subtypes. Importantly, silenced and active rRNA gene subtypes are not intermingled. All silenced rRNA gene subtypes mapped to the nucleolus organizer region (NOR) on chromosome 2 (*NOR2*). All active rRNA gene subtypes mapped to *NOR4*. Using an engineered *A. thaliana* line in which a portion of Col-0 chromosome 4 was replaced by sequences of another ecotype, we show that a major rRNA gene subtype silenced at *NOR2* is active when introgressed into the genome at *NOR4*. Collectively, these results reveal that selective rRNA gene silencing is not regulated gene by gene based on mechanisms dependent on subtle gene sequence variation. Instead, we propose that a subchromosomal silencing mechanism operates on a multimegabase scale to inactivate *NOR2*.

## Abstract (precis)

Given the near sequence identity of ribosomal RNA (rRNA) genes within a species, it is unclear how specific rRNA genes are reproducibly chosen for silencing. Chandrasekhara et al. used *Arabidopsis thaliana* ecotype (strain) Col-0 to identify sequence polymorphisms that differ between active and developmentally silenced rRNA gene subtypes.

Eukaryotes have hundreds, sometimes thousands, of nearly identical copies of ribosomal RNA (rRNA) genes that are transcribed in the nucleolus by RNA polymerase I (Pol I) (Long and Dawid 1980; Gerbi 1985; Hannan et al. 2013; Viktorovskaya and Schneider 2015). These rRNA genes are clustered in long tandem arrays at chromosomal loci known as nucleolus organizer regions (NORs), so named because rRNA transcription and processing cause the nucleolus to form during interphase of each cell cycle (McClintock 1934; Ritossa and Spiegelman 1965; Wallace and Birnstiel 1966). Primary transcripts of rRNA genes, which vary in size from 35S to 48S depending on species, are processed into 18S, 5.8S, and 25S–28S rRNAs that form the catalytic cores of ribosomes, the protein-synthesizing machines of the cell (Turowski and Tollervey 2015).

The number of rRNA genes needed to meet cellular demands for ribosomes and protein synthesis varies depending on cell type, developmental stage, and growth status. Results from studies in yeast, mammals, and plants indicate that rRNA genes are regulated in at least two ways. Coarse control is accomplished via epigenetic regulatory mechanisms that influence the number of rRNA genes that are on or off, thereby regulating their effective dosage (Chen and Pikaard 1997; Santoro and Grummt 2001; Sandmeier et al. 2002; Santoro et al. 2002; Grummt and Pikaard 2003). Fine-tuning is then accomplished by regulating the number of RNA Pol I enzymes engaged in transcription at each active gene (McStay and Grummt 2008; Grummt and Langst 2013).

Within a species, rRNA genes are nearly identical in sequence, so how can rRNA genes be discriminated from one another to dictate which should be expressed and which should be silenced? Hypotheses for the selective expression of rRNA genes in multicellular eukaryotes have favored the idea that the on/off state is somehow determined one gene at a time based on sequence information inherent to each gene. Initially, evidence suggested that specific rRNA genes might be preferentially activated based on sequence differences that altered transcription factor-binding affinities (for review, see Reeder 1985). However, subsequent studies in plants found that differential rRNA gene activity involved selective silencing rather than selective activation (Chen and Pikaard 1997), with no differences in transcription factor affinities detectable using transient expression or cell-free transcription systems (Saez-Vasquez and Pikaard 1997; Frieman et al. 1999). In both plants and mammals, rRNA gene silencing involves cytosine hypermethylation, repressive histone modifications, and noncoding RNAs (Chen and Pikaard 1997; Santoro and Grummt 2001; Santoro et al. 2002, 2010; Zhou et al. 2002; Lawrence et al. 2004; Strohner et al. 2004; Earley et al. 2006; Li et al. 2006; Mayer et al. 2006; Preuss et al. 2008; Pontvianne et al. 2012). Gene sequence differences that affect nucleosome positioning (Felle et al. 2010) or the base-pairing of regulatory noncoding RNAs (Preuss et al. 2008; Santoro et al. 2010) have been proposed as mechanisms to explain how specific rRNA genes might be selectively silenced. In genetic hybrids in which the rRNA genes of the progenitors are differentially expressed, an epigenetic phenomenon known as nucleolar dominance (Tucker et al. 2010), such hypotheses are plausible because the rRNA genes of the progenitor species can differ substantially in their regulatory sequences. However, it is less clear how rRNA genes in nonhybrids can be discriminated from one another when they are nearly identical in sequence.

In this study, we exploited the genetic and genomic resources of *Arabidopsis thaliana* as a model system to investigate how rRNA genes are selected for silencing or activity during vegetative development. By systematically searching for sequence differences that define multiple rRNA gene subtypes in the ecotype (strain) Col-0, determining which of these subtypes are subjected to epigenetic silencing and which are constitutively expressed, and then mapping the chromosomal locations of the various gene subtypes using recombinant inbred populations derived from four genetically distinct ecotypes, we show that selective rRNA gene silencing is not determined based on rRNA gene sequence or subtype. Instead, it is the chromosomal position of rRNA genes at the NOR on chromosome 2 (*NOR2*) that determines whether rRNA genes will be subjected to chromatin-mediated silencing during development.

## Results

### Sequence variation in silenced versus active rRNA genes

**Figure 1.**
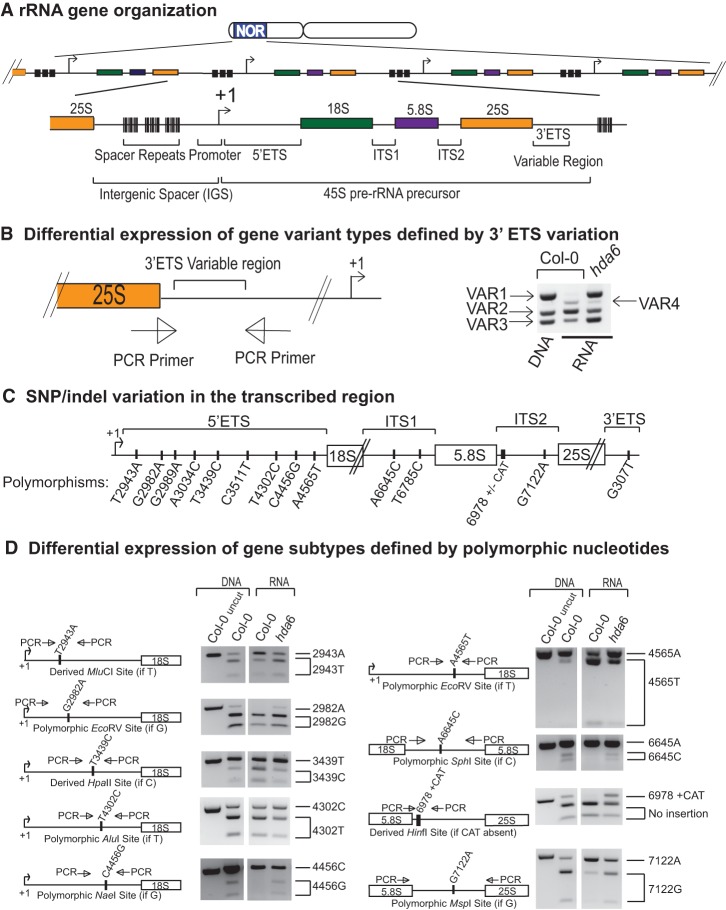
Selective silencing of *A. thaliana* 45S rRNA gene subtypes. (*A*) 45S rRNA genes are repeated in long tandem arrays at NORs located on chromosomes 2 and 4. Each rRNA gene repeat includes a 45S pre-rRNA transcription unit and an intergenic spacer (IGS) that includes the gene promoter. The 5′ and 3′ external transcribed spacer sequences (5′ ETS and 3′ ETS) and internal transcribed spacers (ITS1 and ITS2) are removed during pre-RNA processing. Within the 3′ ETS, a variable region defines rRNA gene types Variant 1 (VAR1) through VAR4. (*B*) Primers flanking the 3′ ETS variable region yield PCR products that define rRNA gene types VAR1, VAR2, VAR3, and VAR4. The gel image at the *right* shows PCR products upon amplification of genomic DNA or cDNA of leaf tissue RNA, comparing wild-type and an *hda6*-null mutant. PCR primer sequences are provided in Supplemental Figure S1. (*C*) Locations of nucleotide polymorphisms that define rRNA gene subtypes. Positions of polymorphic nucleotides are numbered relative to the 45S rRNA gene reference sequence provided in Supplemental Figure S2. Single-nucleotide polymorphism names begin with the nucleotide of the reference sequence, followed by the position in the reference sequence, and ending with the alternative nucleotide at that position. 6978+CAT is a trinucleotide insertion (CAT) relative to the reference sequence. The relative frequencies of the alternative polymorphic nucleotides is provided in Supplemental Figure S3. (*D*) Differential expression of rRNA gene subtypes defined by sequence polymorphisms. The diagrams at the *left* of the gel images show the positions of PCR primers flanking the polymorphic site and the natural or derived restriction endonuclease recognition site that results from the polymorphism. Gel images show PCR products obtained using genomic DNA or RT–PCR in the wild type (Col-0) or *hda6* mutant (in the Col-0 genetic background) following digestion with the relevant restriction endonuclease. Uncut controls are also shown in each case.

In *A. thaliana*, NORs are the most distal genetic loci on the northern ends of chromosomes two and four (*NOR2* and *NOR4*, respectively), where the terminal rRNA genes are capped directly by telomere repeats (Fig. 1A; Copenhaver et al. 1995; Copenhaver and Pikaard 1996a). In the ecotype (strain) Col-0, whose genome provides the primary reference sequence for *A. thaliana* as a species, and in ecotype Ler, pulsed-field gel electrophoresis studies indicated that *NOR2* and *NOR4* consist of ∼350–400 rRNA genes each. With each diploid cell having ∼1400–1600 rRNA genes and each rRNA gene spanning ∼10–10.5 kb, the total rRNA gene content is ∼16 Mb (Copenhaver and Pikaard 1996b).

In the Col-0 ecotype, four rRNA gene types have been identified thus far based on differences within a repetitive region of the external transcribed spacer (ETS) located just 3′ of the 25S rRNA sequences (Figs. 1A,B; Pontvianne et al. 2010). These four gene types are revealed by PCR amplification of genomic DNA using a primer pair that flanks a 3′ ETS variable region (Fig. 1B; primer sequences are provided in Supplemental Fig. S1). Three of the rRNA gene types are abundant (Variant 1 [VAR1], VAR2, VAR3), and one is relatively rare (VAR4). All four classes of rRNA genes are expressed in newly germinated seeds, but, by 10–14 d after germination and throughout the remainder of vegetative development, the VAR1 class, accounting for ∼50% of the total rRNA gene pool, is selectively silenced (Earley et al. 2006, 2010; Pontvianne et al. 2010). However, VAR1 genes fail to be silenced if seeds are germinated on medium containing the cytosine methylation inhibitor 5-aza-2′-deoxycytosine or if plants are defective for repressive chromatin-modifying activities, such as HISTONE DEACETYLASE 6 (HDA6) (see Fig. 1B; Earley et al. 2006, 2010; Pontvianne et al. 2010). Unlike VAR1 genes, which become selectively silenced in emerging leaves of seedlings (Earley et al. 2010), the VAR2, VAR3, and VAR4 rRNA genes are constitutively expressed in leaves (Fig. 1B). However, VAR3 gene expression is elevated in *hda6* mutants (Fig. 1B), suggesting that a subset of VAR3 genes is subjected to chromatin-mediated silencing.

We searched for rRNA gene sequence variation within pre-rRNA transcription units by analyzing short read genome sequence data for potential single-nucleotide polymorphisms (SNPs) relative to a consensus 45S rRNA gene reference sequence (Supplemental Fig. S2). Candidate polymorphisms, supported by multiple independent reads, were examined further using PCR amplification of the region and sequencing of multiple independent clones. Thirteen SNPs and one trinucleotide (CAT) insertion were confirmed (Supplemental Fig. S3). All are located in pre-rRNA sequences removed during processing; namely, within the 5′ ETS, 3′ ETS, ITS1 (internal transcribed spacer 1), or ITS2 (see Fig. 1C). Some nucleotide sequence polymorphisms create natural restriction endonuclease recognition sites, allowing genes that differ with respect to the polymorphism to be discriminated using CAPS (cleaved amplified polymorphic sequence) assays. In a CAPS assay, PCR amplification products are digested with a restriction endonuclease and resolved by agarose gel electrophoresis, allowing digested and undigested PCR products to be visualized (Fig. 1D). Other sequence polymorphisms allowed for derived CAPS (dCAPS) assays (Neff et al. 1998) in which the primer sequence, in conjunction with the polymorphism, contributes to the creation or abolition of a restriction endonuclease recognition site (Fig. 1D, 2943T, 3439C, and indel 6978 polymorphisms). By conducting CAPS or dCAPS assays using genomic DNA as well as cDNA obtained by RT–PCR of leaf RNA, we ascertained the abundance and expression status of rRNA gene subtypes bearing the polymorphic nucleotides. Moreover, by comparing PCR and RT–PCR results for wild-type Col-0 or *hda6*-null mutants, we identified SNPs that are present in gene subtypes that are normally subjected to chromatin-mediated silencing but fail to be silenced in *hda6* mutants (Fig. 1D). Collectively, these assays revealed that SNPs 2943A, 3439C, 4302C, 4565T, and 7122A are present in rRNA genes that are active in leaves of wild-type plants. In contrast, SNPs 2982A, 4456G, and 6645C and insertion 6978+CAT were not detected in the RNA of wild-type plants yet were expressed in the *hda6* mutant, indicating that rRNA genes bearing these polymorphic nucleotides are subjected to chromatin-mediated silencing.

### SNPs and 3′ variable regions can be linked to distinct rRNA gene haplotypes

**Figure 2.**
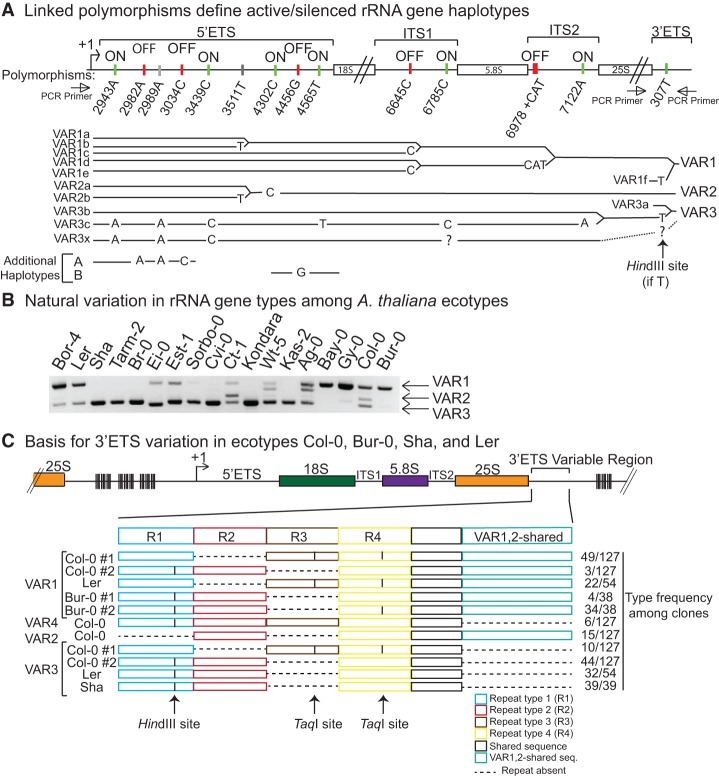
rRNA gene subtype variation. (*A*) Expression status of gene subtypes represented by nucleotide polymorphisms and linkage of polymorphisms to define VAR class haplotypes. Polymorphic nucleotides present in expressed rRNA genes, based on RT-CAPS/dCAPS assays, are labeled “on,” whereas polymorphic nucleotides not detected in RNA of wild-type plants are labeled “off.” Polymorphisms associated with silent as well as active haplotypes are denoted by gray vertical tick marks. In the tree diagram, solid black lines denote identity to the reference sequence. Nucleotide polymorphisms relative to the reference sequence are shown and aligned with the diagram at the *top* that provides the position number. Dashed lines indicate undefined sequence intervals. Susceptibility of rRNA gene subtypes to digestion by HindIII, due to polymorphism 307T, is shown in Supplemental Figure S4. (*B*) Natural variation in rRNA gene variant type content in diverse *A. thaliana* ecotypes. The gel image shows PCR products obtained using the primer pair flanking the 3′ ETS variable region (see Fig. 1B). (*C*) Basis for 3′ ETS variation in *A. thaliana* ecotypes Col-0, Bur-0, Ler, and Sha. Independent clones of PCR products obtained upon amplification of the 3′ ETS variable region of Col-0, Bur-0, Ler, and Sha rRNA genes were sequenced (see Supplemental Fig. S5). The diagrams depict the presence or absence (dashed lines) of repeat motifs shown in different colors. The number of clones representing each subtype is provided at the *right* of the figure.

In an effort to link the nucleotide polymorphisms described in Figure 1, C and D, with the differences in the 3′ ETS variable region that define the VAR1, VAR2, VAR3, and VAR4 gene types (see Fig. 1A,B), long PCR products, including some that extend ∼8.5 kb from the transcription initiation site to the 3′ end of the 3′ ETS variable region, were cloned and sequenced. The results are summarized in Figure 2A. SNPs combined with the 3′ ETS variable region define at least six distinct VAR1 haplotypes (VAR1a, VAR1b, VAR1c, VAR1d, VAR1e, and VAR1f), at least two VAR2 haplotypes (VAR2a and VAR2b), and at least three VAR3 haplotypes (VAR3a, VAR3b, and VAR3c). Haplotypes defined by SNPs that we were unable to link to the 3′ ETS variable region are VAR3x and haplotypes A and B (Fig. 2A). By combining the data of Figure 1 (identifying SNPs present in expressed or silenced rRNA genes) with the haplotype assembly results of Figure 2A, we deduce that active rRNA gene haplotypes include VAR2a and/or VAR2b (containing SNP 4302C), VAR3c (containing SNPs 2943A, 3439C, 4565T, 6785C, and 7122A), and VAR4. Conversely, silenced rRNA gene haplotypes include VAR1c, VAR1d, and VAR1e (containing SNP 6645C and indel 6978) and haplotypes A and B.

A SNP within the variable region (307T) produces a HindIII site in VAR3b,c genes (and likely in VAR3x genes) as well as in VAR1f and VAR4 genes. This SNP is absent in VAR3a genes, allowing the estimation that VAR3a genes account for ∼10% of the total VAR3 pool based on the amount of full-length VAR3 PCR product that is resistant to HindIII digestion (Supplemental Fig. S4). We tested the relative expression status of VAR3 subtypes by using PCR primers flanking polymorphic position 307 (see Fig. 2A, diagram) to amplify, clone, and sequence cDNAs of leaf RNA obtained by RT–PCR. Among 53 VAR3 cDNA clones obtained using RNA of wild-type plants, 100% had the 307T SNP (and thus a HindIII site). No VAR3a cDNA clones bearing the 307G reference sequence were obtained. However, upon testing RNA of the *hda6* mutant, five of the 58 VAR3 clones were of the VAR3a subtype, which is similar to the estimated relative abundance of the VAR3a subtype (∼10%). These results indicate that haplotype VAR3a is subjected to chromatin-mediated silencing, like VAR1 haplotypes but unlike the VAR3c haplotype.

### rRNA gene subtype natural variation

Having identified polymorphisms that define rRNA gene subtypes of ecotype Col-0 that differ in expression state, we sought to determine whether differentially expressed subtypes are interspersed or segregated from one another. To test the feasibility of a genetic mapping approach, we evaluated subtype variation in *A. thaliana* by using PCR amplification of the 3′ ETS variable region with genomic DNA from 17 ecotypes in addition to Col-0 (Fig. 2B). The results suggested that each ecotype shares one or more rRNA gene subtypes present in Col-0 (see also Abou-Ellail et al. 2011). Sequencing of PCR products confirmed that Ler and Bur-0 have VAR1 genes, and Ler and Sha have VAR3 genes like those of Col-0 (Fig. 2C; Supplemental Fig. S5), indicating that these rRNA gene types existed prior to the divergence or geographical separation of the ecotypes. Col-0 has at least one subtype of VAR1 (Var1f) that has the 307T SNP (also present in 90% of VAR3 genes), creating a HindIII site, but this subtype is rare such that HindIII digestion has no perceptible effect on the abundance of VAR1 3′ ETS PCR products (Supplemental Fig. S4). In ecotype Ler, we detected only one version of the VAR1 3′ ETS region, which is identical in sequence to Col-0 VAR1 subtypes that lack the HindIII site. In contrast, Bur-0 has two VAR1 subtypes that have the HindIII site but differ with respect to a SNP that creates/abolishes a TaqI site in repeat 4 (Fig. 2C). Ler and Sha have VAR3 genes that are identical in sequence to the VAR3b or VAR3c subtype of Col-0 in the 3′ ETS region. VAR2 and VAR4 rRNA gene types were not detected in Bur-0, Sha, or Ler but may be present in other ecotypes, such as Wt-5, Ag-0, or Ct-1 (see Fig. 2B).

### Mapping rRNA gene subtypes to NOR2 or NOR4

Natural variation in rRNA gene subtype content enabled us to map the chromosomal positions of Col-0 rRNA gene subtypes using recombinant inbred lines (RILs). RILs are generated by first crossing two ecotypes (or strains) that differ with respect to a trait of interest. An F1 progeny is then allowed to self-pollinate, generating seeds for thousands of F2 siblings that differ as a consequence of stochastic meiotic recombination events. Hundreds of F2 individuals are grown, and single F3 seeds from each F2 plant are germinated and grown. This process of single-seed descent is repeated for multiple generations. By the F8 generation, individual RILs have a >99% probability of being homozygous at any given locus while having genomes that are a patchwork of chromosomal segments derived from each progenitor (Koornneef et al. 2004; Broman 2005). By monitoring the segregation of a trait of interest among the RIL population and determining how the trait is linked to adjacent ecotype-specific chromosomal markers, the map position of the trait is determined.

**Figure 3.**
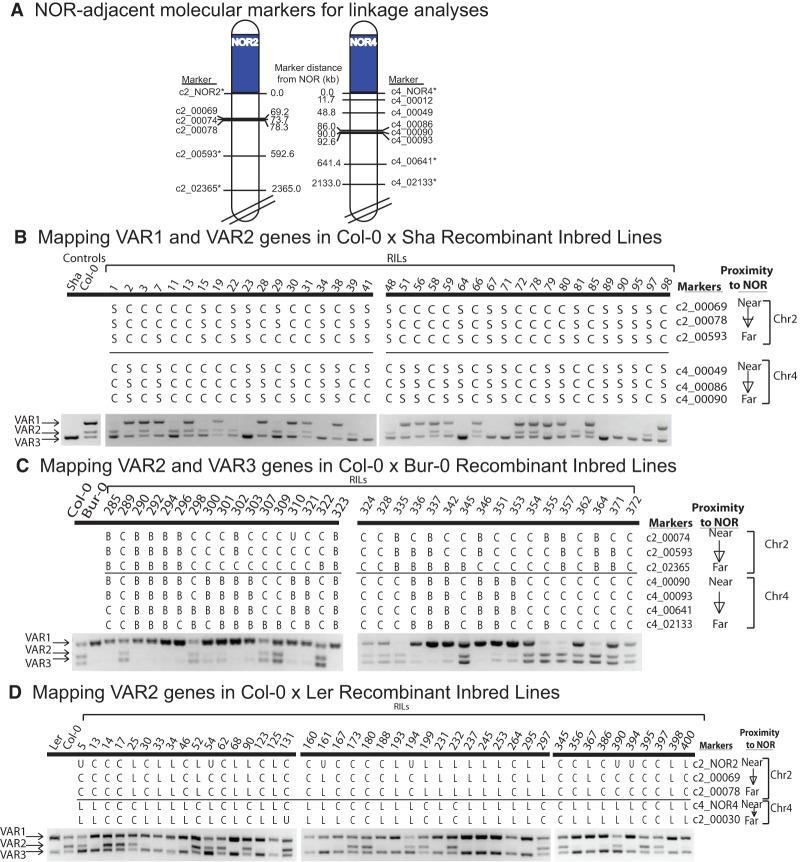
Mapping NOR associations of VAR1-, VAR2-, and VAR3-type rRNA genes using RILs. (*A*) Positions of molecular markers used for linkage analyses. The tops of chromosomes 2 and 4 are diagrammed, with NORs colored blue. Positions of sequence polymorphisms that yield ecotype-specific CAPS markers are shown, along with their distances (in kilobases) from the NORs. Each marker name begins with c2 or c4 to denote chromosome 2 or 4, followed by a number denoting the distance (in kilobases) from the NOR. An asterisk denotes a marker identity determined by Simon et al. (2008). (*B–D*) Segregation of VAR1, VAR2, or VAR3 in Col-0 × Sha, Col-0 × Bur-0, or Col-0 × Ler RILs. In each panel, the *left* lanes show the PCR products obtained using genomic DNA of the parental ecotypes and a primer pair that flanks the 3′ ETS variable region (see Fig. 1B). The remaining lanes show PCR products using the indicated RILs. RILs were genotyped for the indicated markers, which are arranged according to their proximity to *NOR2* or *NOR4*. Markers derived from one or the other ecotype are indicated by the first letter of that ecotype's name. “U” denotes unscored, indicating ambiguity in the marker determination. Analyses of additional Col × Sha and Col × Bur-0 RILs are provided in Supplemental Figures S6 and S7, respectively.

To map the chromosomal positions of Col-0 VAR1, VAR2, and VAR3 rRNA gene types, we made use of Col-0 × Sha, Col-0 × Bur-0, and Col-0 × Ler RILs (Lister and Dean 1993; Simon et al. 2008). Segregation of the Col-0-specific rRNA gene classes within the RIL populations was scored, as were ecotype-specific molecular markers located in the chromosomal regions adjacent to NOR2 or NOR4 (Fig. 3A). DNA purified from 81 Col-0 × Sha RILs was tested by PCR for the presence of VAR1 or VAR2 genes, which are absent in Sha and thus inherited solely from Col-0 (Fig. 3B; see also Supplemental Fig. S6). In 19 lines in which genotyping indicated that the top of chromosome 2 was derived from Sha and that the top of chromosome 4 was derived from Col-0, VAR2 was detected in 18, allowing the deduction that VAR2 genes map to Col-0 NOR4. Twenty-six Col-0 × Sha RILs had the top of chromosome 2 derived from Col-0 and the top of chromosome 4 derived from Sha (Fig. 3; Supplemental Fig. S6). Among these 26 lines, all 26 had Col-0-specific VAR1 genes, indicating that VAR1 genes are located within NOR2.

VAR3 is absent in ecotype Bur-0, which allowed the mapping of Col-0 VAR3 genes using Col-0 × Bur-0 RILs (Fig. 3C; Supplemental Fig. S7). In 17 of the 80 lines tested, genotyping indicated that the top of chromosome 2 is derived from Col-0, and the top of chromosome 4 is from Bur-0. Each of these 17 lines yielded modest VAR3 PCR signals, indicating that some VAR3 genes are located at NOR2 of Col-0, and none yielded significant VAR2 signals, consistent with VAR2 genes mapping to NOR4. Thirteen lines had the reciprocal genotype, with the top of chromosome 2 derived from Bur-0, and the top of chromosome 4 derived from Col-0. Eleven of these lines yielded strong signals for both VAR2 and VAR3. Collectively, these results show that the majority of VAR3 rRNA genes in Col-0 cosegregate with VAR2 genes at NOR4, but a subset of VAR3 genes is located at NOR2.

Ecotypes Col-0 and Ler have VAR1 and VAR3 genes but differ with respect to VAR2, allowing VAR2 genes to be mapped in yet a third RIL population (Fig. 3D). VAR2 genes are present in all six RILs that have Col-0 markers at the top of chromosome 4 and Ler markers at the top of chromosome 2. Conversely, 12 of the 16 RILs that have the top of chromosome 2 derived from Col-0 and the top of chromosome 4 from Ler lack significant VAR2 signals. Together with the Col-0 × Sha and Col-0 × Bur-0 RIL mapping results, these data show that VAR2 genes map primarily to NOR4.

**Figure 4.**
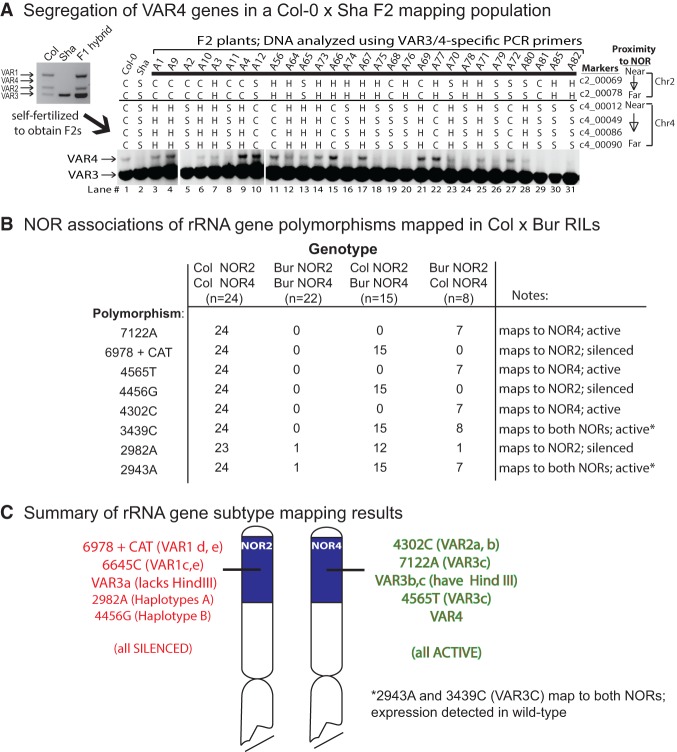
NOR associations of VAR4 and rRNA gene subtypes defined by nucleotide polymorphisms. (*A*) Segregation of VAR4 genes in a subset of Col-0 × Sha F2 individuals. The two *left* lanes show controls in which PCR amplification of Col-0 or Sha genomic DNA was conducted using a primer pair that amplifies only VAR3 or VAR4 genes and not VAR1 or VAR2 genes. F2 individuals were scored with respect to markers adjacent to *NOR2* or *NOR4*. Markers are scored as C or S if homozygous for Col-0- or Sha-specific polymorphisms and H if heterozygous. The specificity of the PCR amplification procedure and a summary of the mapping results is in Supplemental Figure S8. (*B*) NOR associations of rRNA genes bearing eight distinct nucleotide polymorphisms mapped using Col-0 × Bur-0 RILs. The frequency of each polymorphism in the various genotypes and the deduced map positions and expression status (in leaf RNA) of genes bearing the polymorphisms are summarized. Active gene subtypes marked with an asterisk map to both NORs but may be expressed at only one NOR. The raw PCR analyses whose results are summarized in the table are in Supplemental Figure S9. Analogous PCR analyses using Col × Sha RILS, revealing the map positions of VAR3a and SNP 6645C, are in Supplemental Figure S10. (*C*) Summary of mapping results showing the deduced NOR associations of rRNA gene subtypes that are active in leaves (shown in green) versus those subtypes that are selectively silenced during development (shown in red).

VAR4 genes are relatively low in abundance compared with VAR1, VAR2, or VAR3 genes such that they are detected at only trace levels upon PCR amplification of genomic DNA (see Fig. 1B). However, VAR4 gene transcripts are readily detected by RT–PCR, indicating that VAR4 genes are expressed in leaves at disproportionately high levels (see Fig. 1B). Using a PCR primer pair that selectively amplifies the 3′ ETS variable regions of VAR3 and VAR4 rRNA genes, but not VAR1 or VAR2 genes, thus improving VAR4 gene detection using genomic DNA, cosegregation of VAR4 genes with NOR-adjacent markers was assayed in a Col-0 × Sha F2 mapping population of 92 individuals (Fig. 4A; the PCR primer specificity is shown in Supplemental Fig. S8). VAR4 genes are absent in Sha; thus, their Col-0 NOR associations are easily ascertained. In F2 individuals that inherited NOR4 from Col-0, VAR4 genes are present (e.g., see F2 individuals A12 and A72 that are homozygous for NOR4 of Col-0 and NOR2 of Sha). Conversely, VAR4 genes are not detected in lines in which NOR2 was inherited from Col-0 (e.g., F2 individuals A2, A11, A68, and A81). Collectively, these data indicate that VAR4 genes are located at NOR4.

We next mapped the genomic locations of rRNA genes bearing the various SNPs that define active versus silenced rRNA gene subtypes (see Fig. 1D). For these analyses, DNA isolated from RILs was tested using CAPS or dCAPS assays as in Figure 1D. NOR association mapping data for polymorphic nucleotides mapped in Col-0 × Bur-0 RILs are shown in Supplemental Figure S9 and summarized in Figure 4B. Collectively, these analyses showed that SNPs that map to NOR2 of Col-0 include insertion 6978+CAT (present in VAR1d,e), 2982A (present in haplotype A), and 4456G (present in haplotype B). Importantly, none of these NOR2-linked polymorphisms are detected in RNA isolated from leaves of wild-type plants, but all are expressed if silencing is abrogated in the *hda6* mutant (see Fig. 1D). In contrast, SNPs that map to NOR4 include 4302C (VAR2 a/b), 4565T (present in VAR3c), and 7122A (present in VAR3c), all of which are expressed in both wild-type plants and *hda6* mutants. Two of the SNPs that are present in the VAR3c haplotype mapped to both NORs (2943A and 3439C), whereas other SNPs present in VAR3c mapped exclusively to NOR4 (4565T and 7122A). These mapping data are explained by deduced haplotype VAR3x, which has the 2943A, 2989A, and 3439C SNPs but not the 4565T or 7122A SNPs (see Fig. 2A). We further deduce that the VAR3x haplotype maps to NOR2.

To determine the chromosomal locations of VAR3a genes as well as genes bearing SNP 6645C, both of which are silenced in an *hda6*-dependent manner in Col-0, we made use of Col-0 × Sha RILs (Supplemental Fig. S10). RIL genomic DNA was subjected to PCR amplification of the 3′ ETS variable region followed by digestion with HindIII (for VAR3a) or SphI (for SNP 6645C) and agarose gel electrophoresis. Virtually all Sha rRNA gene PCR products are cut by HindIII, as are Col-0 Var3b and VAR3c genes (see Fig. 2C; Supplemental Fig. S4), such that only the PCR products of VAR3a genes inherited from Col-0 are left uncut after HindIII digestion. SNP 6645C is absent in Sha rRNA genes but creates an SphI site in those Col-0 genes that have the SNP. Collectively, the CAPS analyses revealed that VAR3a genes and genes bearing SNP 6645C both map to NOR2 of Col-0 such that both subtypes are present in all 26 of the RILs in which NOR2 is derived from Col-0 and NOR4 is derived from Sha (Supplemental Fig. S10).

Figure 4C shows a graphical summary of all of the mapping results. Importantly, all rRNA gene subtypes that are subjected to HDA6-mediated silencing map to NOR2. Conversely, all of the rRNA gene subtypes that are expressed in leaves map to NOR4.

### Bacterial artificial chromosome (BAC) clones reveal variable VAR1–VAR3 interspersion patterns

**Figure 5.**
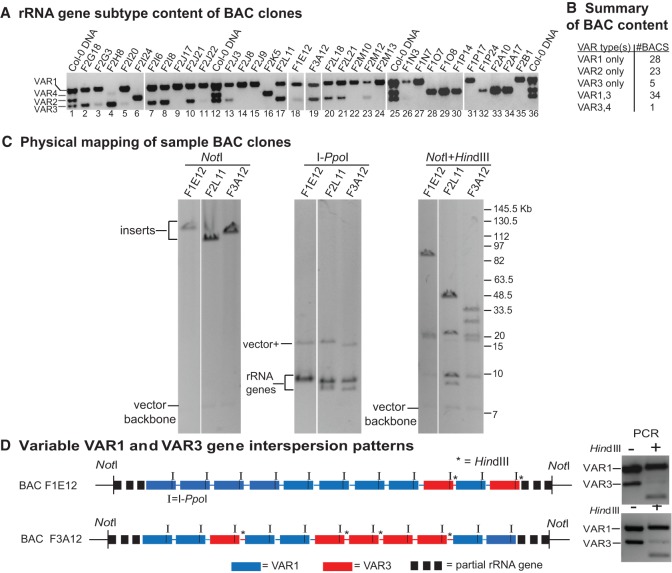
rRNA gene variant type content within BAC clones. (*A*) PCR detection of rRNA gene variant types present within a subset of 91 Col-0 genomic DNA BAC clones known to carry rRNA genes. Lanes *1*, *12*, *25*, and *36* show PCR products obtained using the primer pair flanking the 3′ ETS variable region and genomic DNA; all other lanes show PCR products obtained using DNA isolated from the indicated BAC clones. (*B*) Summary of rRNA gene types present within 91 BAC clones tested. (*C*) Examination of VAR1 and VAR3 interspersion patterns in three BAC clones. NotI digestion liberates cloned inserts of ∼120–130 kb from the ∼7-kb vector backbone, indicating that the clones potentially have ∼12–13 rRNA genes. I-PpoI recognizes a 15-base-pair recognition sequence within rRNA genes, generating unit-length rRNA gene digestion products of ∼10 kb. HindIII does not cut the majority of VAR1-type rRNA genes but does cut most VAR3 genes such that double digestion with NotI and HindIII provides insight into VAR1–VAR3 interspersion patterns. (*D*) Diagrammatic models for VAR1 and VAR3 interspersion in BAC clones F1E12 and F3A12 that are consistent with the restriction digestion analyses shown in *B*. Other models are also possible. The PCR analyses at the *right* of the diagrams suggest that one or more of the VAR3 genes in clone F3A12 are of the VAR3a type not cut by HindIII.

VAR3 genes map to both NORs; thus, we looked for evidence that they might be interspersed with VAR1 genes at NOR2 and/or VAR2 genes at NOR4 by analyzing 91 Col-0 BAC clones known to contain rRNA genes (Fig. 5; see also Supplemental Fig. S11). The BAC clones were found to have inserts averaging ∼100 kb, based on restriction endonuclease digestion and pulsed-field gel electrophoresis, and thus contained, on average, ∼10 rRNA genes each. Twenty-three BAC clones tested positive for VAR2 rRNA genes, but none yielded VAR3 PCR products, suggesting that VAR2 and VAR3 genes mostly (if not entirely) occupy different portions of NOR4 (Fig. 5A,B). In contrast, VAR1 and VAR3 genes are present together in 34 BAC clones, suggesting that these subtypes are interspersed at NOR2. VAR1 genes typically lack HindIII sites (see Supplemental Fig. S4), whereas the majority of VAR3 genes are cut by HindIII (i.e., the VAR3b or VAR3c subtypes). Thus, HindIII digestion provided insight into VAR1–VAR3 interspersion patterns in BAC clones that contain both gene types. These analyses, conducted using contour-clamped homogenous electric field (CHEF) gel electrophoresis, revealed that VAR3 genes are interspersed with VAR1 genes at irregular intervals, as shown in Figure 5C for BAC clones F1E12, F2L11, and F3A12. For each clone, NotI digestion releases cloned inserts of ∼110–130 kb from the vector backbone, and digestion with I*-*PpoI, which cuts once in each rRNA gene, releases unit-length rRNA gene fragments of ∼10 kb, indicating that the cloned inserts are composed exclusively of rRNA gene repeats (Fig. 5C). Double digestion with NotI and HindIII yielded unique digestion patterns for each of the three BACs, indicating that VAR1 and VAR3 genes are interspersed at irregular intervals, as illustrated in Figure 5D. VAR1 genes can also be present in arrays that are not interrupted by VAR3 genes, as in 28 BAC clones that consisted of VAR1 genes only. Collectively, our genetic mapping experiments, combined with the BAC clone analyses, indicate that HindIII-containing VAR3 genes are interspersed sporadically among the more abundant VAR1 genes at NOR2. This sporadic interspersion pattern results in HindIII sites that are sometimes located in adjacent genes or in genes that can be tens or hundreds of kilobases apart, thus providing a molecular explanation for the large, ecotype-specific HindIII fragments that were used to map NOR2 to the top of chromosome 2 in the studies of Copenhaver et al. (1995). Because VAR3c genes genetically map to NOR4 and because SNPs 7122A and 4565T are absent in three BAC clones in which VAR1 and VAR3 genes are interspersed, we deduce that the HindIII-bearing VAR3 genes interspersed with VAR1 at NOR2 are of the VAR3b or VAR3x subtypes.

### VAR1 genes are expressed if located at *NOR4*

**Figure 6.**
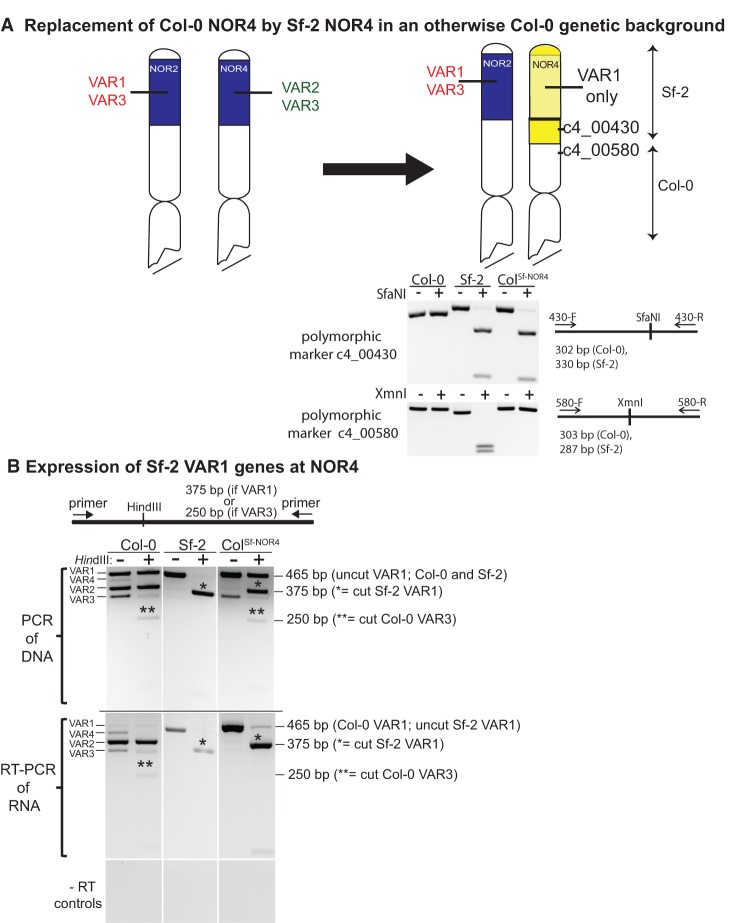
VAR1-type rRNA genes can be expressed when located at *NOR4*. (*A*) Diagrams of *NOR2* and *NOR4* in a plant line (termed Col^Sf-NOR4^) in which Sf-2 *NOR4* and adjacent sequences were introgressed into the Col-0 genetic background. The PCR data show genotyping analyses using polymorphic markers c4_00430 and c4_00580 that indicate that the recombination site joining Col-0 to Sf-2 sequences occurs within the interval located 430–580 kb from *NOR4*. The rRNA genes of Sf-2 *NOR4* are all of the VAR1 type. (*B*) Sf-2-derived VAR1 genes are expressed at chromosome 4. The *top* set of gel images shows PCR products obtained using genomic DNA of Col-0, Sf-2, or the introgression line Col^Sf-NOR4^ with or without digestion by HindIII. VAR1 genes of Col-0 are not cut by HindIII, whereas Sf-2 VAR1 genes are cut, allowing the VAR1 genes of the two ecotypes to be discriminated in the Col^Sf-NOR4^ line. The *middle* panel shows the expression status of the different VAR types present in Col-0, Sf-2, or the introgression line Col^Sf-NOR4^, determined using RT–PCR with the same primers used for DNA analyses. The *bottom* panel shows controls in which reverse transcriptase was omitted from RT–PCR reactions.

Our genetic mapping and RNA expression assays show that rRNA gene subtypes silenced in leaves of ecotype Col-0 are located at *NOR2*, whereas active rRNA genes map to *NOR4*, suggesting that chromosomal position rather than rRNA gene subtype may determine rRNA gene expression status. A prediction of this hypothesis is that VAR1 genes, the predominant subtype present at *NOR2*, would not be silenced if they were located at *NOR4*. As a test of this hypothesis, we exploited an *A. thaliana* line generated to study cold-responsive flowering in plants carrying the *FRIGIDA* (*FRI*) gene of ecotype Sf-2 (Lee and Amasino 1995). This line was initially generated by crossing Sf-2 with Col-0 followed by recurrent backcrossing of progeny to Col-0 for six generations, with selection for the Sf-2 *FRI* gene in each generation. Several additional generations of backcrossing were conducted in the Scott Michaels laboratory. Because *FRI* is located only ∼280 kb from *NOR4*, we tested whether Sf-2 *NOR4*, which is composed entirely of VAR1 rRNA genes, might also have been introgressed into the Col-0 background, replacing Col-0 *NOR4*. Indeed, this is the case. In the introgression line, tests for molecular markers adjacent to *NOR4* show that the top of Col-0 chromosome 4 is replaced by corresponding Sf-2 sequences, with the recombination breakpoint occurring within the interval defined by markers c4_00430 and c4_00580, located 430 kb and 580 kb, respectively, from the centromere-proximal boundary of *NOR4* (Fig. 6A). Moreover, PCR tests show that VAR2 and VAR4 class rRNA genes, which are indicative of *NOR4* of Col-0, are absent in the introgression line (referred to here as Col^Sf-NOR4^) due to replacement of the entire NOR by *NOR4* of Sf-2 (Fig. 6B). As a result, PCR amplification of Col^Sf-NOR4^ genomic DNA results in highly abundant VAR1 gene amplification products derived from both NORs and less abundant VAR3 products derived from Col-0 *NOR2* (Fig. 6B). Importantly, virtually all VAR1 genes of Sf-2 carry the 307T SNP that creates a HindIII site, allowing these genes to be discriminated from the VAR1 genes of Col-0, almost all of which carry the 307G SNP (see Fig. 6B, top panels). Approximately half of the Col-0 VAR3 genes at NOR2 likewise carry the 307T SNP and are cut by HindIII (Fig. 6B).

We tested the expression status of the different rRNA gene types present in Col-0, Sf-2 and Col^Sf-NOR4^ by RT–PCR (Fig. 6B, middle panels). In Col-0, VAR2, VAR3, and VAR4 are expressed at significant levels, whereas VAR1 genes are expressed at only trace levels. In Sf-2, whose rRNA genes are almost entirely of the VAR1 class, VAR1 genes are highly expressed, with resulting cDNAs sensitive to HindIII digestion. In the introgression line Col^Sf-NOR4^, VAR1 genes are also highly expressed, with the majority of the resulting cDNAs being cut by HindIII, indicating that these expressed VAR1 genes are those derived from Sf-2 and located at *NOR4*. In contrast, the VAR1 (and VAR3) genes of Col-0 located at *NOR2* are detected at only trace levels. Collectively, these data indicate that VAR1 genes are capable of being highly expressed in the Col-0 genetic background if they are located at *NOR4* or, alternatively, are not located at *NOR2*.

## Discussion

We determined the NOR associations of rRNA gene variant types VAR1, VAR2, VAR3, and VAR4 as well as rRNA gene subtypes bearing a dozen distinct nucleotide polymorphisms. Importantly, those rRNA gene subtypes that are silenced in a HDA6-dependent manner during early vegetative development all map to *NOR2*. In contrast, rRNA gene subtypes that are not subjected to silencing and remain expressed in leaves all map to *NOR4*. Collectively, these data suggest that the silencing events that result in the inactivation of approximately half of all rRNA genes during development do not act on individual rRNA genes based on sequence differences that allow them to be discriminated from other rRNA genes. Indeed, the sequence variation among rRNA genes is subtle, often consisting of SNPs located in regions of the pre-rRNA that are removed during processing or caused by variation in the number of imperfect repeats within the 3′ external transcribed region. None of the variation is detected in the vicinity of the promoter or in regions suspected to play roles in transcriptional regulation.

The only relatively long stretches of contiguous sequence variation that occurs among rRNA gene subtypes are present in the repetitive variable region of the 3′ ETS that defines variant classes VAR1, VAR2, VAR3, and VAR4. However, two lines of evidence indicate that this variation cannot explain whether a given variant type will be constitutively expressed or subjected to silencing. One line of evidence is that VAR3 genes occur at both NORs, with some VAR3 genes being active, and some being silenced. The silenced VAR3 genes include the VAR3a subtype and VAR3b or VAR3x subtypes that map to NOR2. We deduce that the HindIII-containing VAR3 rRNA genes that are interspersed with VAR1 genes at NOR2, most likely VAR3b (and/or possibly VAR3x), are also silenced. In contrast, the majority of VAR3 rRNA genes is present at NOR4 in abundance similar to that of VAR2 genes and is expressed. Genetic mapping of polymorphic nucleotides indicates that these latter VAR3 genes are those of the VAR3c subtype. The increase in VAR3 expression observed in *hda6* plants is presumably due to the failure to silence the VAR3a, VAR3b, and VAR3x genes interspersed with the VAR1 genes at NOR2. A second line of evidence arguing against gene type dictating expression status is that VAR1 type genes can be active when present at NOR4, as in the introgression line Col^Sf-NOR4^, yet are silenced when at NOR2.

**Figure 7.**
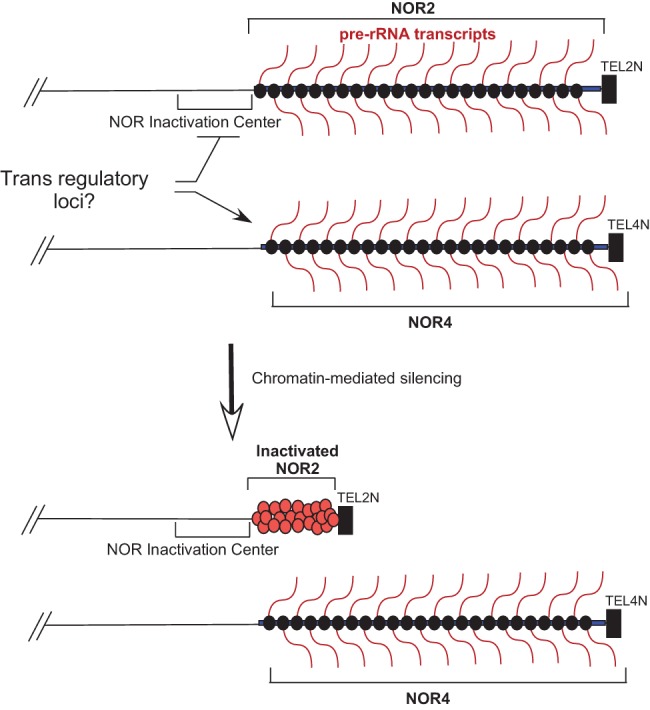
Model for rRNA gene dosage control via selective NOR inactivation. (*Top*) In *A. thaliana* Col-0, all rRNA gene types are expressed in seedlings immediately after germination at both *NOR2* and *NOR4*. However, by 10–14 d of post-embryonic development, rRNA genes located at *NOR2* are selectively silenced via repressive chromatin modifications typical of condensed heterochromatin. (*Bottom*) This includes HDA6-mediated histone deacetylation. Because the two NORs are so similar, including associated telomeres of similar size and rRNA gene numbers and rRNA genes of nearly identical sequence, we postulate that a region adjacent to *NOR2* may be critical for initiating the selective silencing process, which then spreads toward the end of the chromosome. Unlinked regulatory loci may act on the NORs in *trans*, negatively regulating *NOR2* and possibly positively regulating *NOR4*.

We propose that NOR2 is inactivated as a single regulatory locus (Fig. 7), thus accounting for the silencing of ∼50% of all rRNA genes during early post-embryonic vegetative development. The hypothesis that regulation occurs at the NOR level is consistent with prior results from our studies of nucleolar dominance in hybrids of *A. thaliana* × *Arabidopsis lyrata*. In these hybrids, *A. thaliana* rRNA genes are selectively silenced, and *A. lyrata* rRNA genes are preferentially expressed. Importantly, full-length *A. thaliana* rRNA transgenes inserted at ectopic locations in the *A. thaliana* genome outside of the NORs escaped silencing in the interspecific hybrids (Lewis et al. 2007), suggesting that localization within a NOR is important for silencing. Silencing at the level of whole NORs may also be a chromosomal phenomenon common to other multicellular eukaryotes. For instance, in most human cells, only a subset of the NORs located on the five acrocentric chromosomes is associated with the Pol I transcription machinery, indicating that active and inactive rRNA genes are present on different chromosomes (Roussel et al. 1996; McStay and Grummt 2008).

How might NORs be discriminated from one another? In *Arabidopsis*, our previous physical mapping studies using pulsed-field gels indicated that both NORs are composed entirely of head-to-tail rRNA gene repeats oriented in the same direction, without interruption by non-rRNA gene sequences (Copenhaver et al. 1995; Copenhaver and Pikaard 1996a,b). Therefore, it seems unlikely that a putative NOR regulatory control center could be embedded within a NOR. Physical mapping studies also indicated that the terminal rRNA genes of NOR2 and NOR4 are capped by telomere repeats that are added directly to rRNA gene sequences without the presence of unique subtelomeric sequences that might distinguish the two NORs (Copenhaver and Pikaard 1996a). For these reasons, we suspect that sequences adjacent to the NORs on their centromere-proximal sides are likely important for the selective silencing of NOR2 (Fig. 7). Interestingly, the region immediately flanking NOR2 is composed of transposable elements and transposon remnants that extend for ∼60 kb before the first protein-coding genes are encountered. This region is characterized by heavy cytosine methylation and histone post-translational modifications indicative of condensed, transcriptionally repressed heterochromatin. In contrast, the region flanking NOR4 has few transposon-related sequences, with active protein-coding genes occurring within ∼3 kb of the NOR boundary. Thus, one possibility is that NOR2 silencing results from heterochromatin formation that is initiated within the transposon-rich region adjacent to the NOR on the centromere-proximal side and then spreads 4 million base pairs toward the telomere, thereby condensing and inactivating the rRNA genes located within the NOR. With the advent of new tools for chromosome engineering, testing this hypothesis may be feasible.

## Materials and methods

### Plant material and growth

The *A. thaliana* ecotypes, obtained from the *Arabidopsis* Biological Resource Center (ABRC; http://abrc.osu.edu), were as follows: Col-0 (stock no. CS22681), Sha (stock no. CS22690), Bur-0 (stock no. CS22679), C24 (stock no. CS22680), Tarm-2 (stock no. CS22691), Ler-1 (stock no. CS22686), Ag-0 (stock no. CS936), Kas-2 (stock no. CS1264), Wt-5 (stock no. CS1612), Kondara (stock no. CS6175), Ct-1 (stock no. CS1094), Cvi-0 (stock no. CS902), Sorbo (stock no. CS931), Est-1 (stock no. CS6701), Gy-0 (stock no. CS1216), Ei-2 (stock no. CS6689), Br-0 (stock no. CS6626), Bay-0 (stock no. 0CS954), and Bor-4 (stock no. CS22677). RIL population Col-0 × Ler (stock no. CS1899) was also obtained from the ABRC. RIL core population sets Col-0 × Sha and Col-0 × Bur-0 were obtained from Institut Nationale de la Recherche Agronomique (INRA; http://dbsgap.versailles.inra.fr/vnat). All plants were grown in a greenhouse.

### DNA isolation

Two-week-old individual plant leaf tissue was incubated for 10 min at 99°C in extraction buffer (200 mM Tris-HCl at pH 8.0, 250 mM NaCl, 25 mM EDTA, 0.5% SDS). Genomic DNA from the supernatant was recovered by ethanol precipitation and resuspended in 40 µL of Tris-HCl (pH 8.0).

### RNA isolation

Four-week-old leaf tissue of individual plants was frozen in liquid nitrogen and ground into a fine powder. An RNeasy plant minikit (Qiagen) was used to extract total RNA from ground powder. Total RNA was then treated with Turbo DNA-free kit (Invitrogen) to eliminate contaminating DNA.

### PCR assays

For PCR of genomic DNA, PCR amplification was conducted using 20 cycles of 30 sec at 94°C, 30 sec at 55°C–60°C, and 60 sec at 72°C. For RT–PCR, the reverse transcription reaction was performed using 1.5 µg of total RNA and SuperScript III reverse transcriptase (Invitrogen). One microliter (∼100 ng) of reverse transcription product was then used for PCR amplification of rRNA variable regions or an actin control using 26 cycles of 30 sec at 94°C, 30 sec at 56°C, and 60 sec at 72°C. Primers sequences are in Supplemental Fig. S1. PCR and RT–PCR products were resolved by electrophoresis on 2%–2.5% agarose gels in TBE buffer.

### Sequencing of rRNA gene intervals

Genomic DNA extracted from 2-wk-old leaf tissue was used to amplify the 3′ ETS region using primers described above. The amplified region was then cloned into pGEM-T Easy cloning vector (Promega). An Applied Biosystems 3730 automated sequencing system and BigDye Terminator v3.1 cycle sequencing kit (Applied Biosystems) were used to sequence each clone. Sequences were analyzed and aligned using Geneious software (http://www.geneious.com).

### Generation of genetic markers adjacent to *NOR2* and NOR4

Using the Salk Institute Genomics Analysis Laboratory's 1001 Genomes sequencing data, we identified SNPs between parental *A. thaliana* accessions (ecotypes) used for the three RIL populations that we analyzed (http://signal.salk.edu/atg1001/index.php). CAPS assays were generated to distinguish parent-specific markers in each RIL. Markers were named to denote their location on chromosome 2 or chromosome 4 (c2 or c4) and their distance (in kilobases) from the NORs. PCR amplification conditions for CAPs markers were 35 cycles of 30 sec at 94°C, 30 sec at 55°C, and 60 sec at 72°C. Twenty microliters of PCR amplification was then digested with the indicated restriction enzyme according to conditions suggested by New England Biolabs. CAPS products were resolved by electrophoresis on a 2% TBE and agarose gel.

### CHEF gel analyses of BAC clones

BACs containing 45S rRNA genes, identified in the BAC library of Mozo et al. (1998), were obtained from the *Arabidopsis* Biological Resource Center. DNA from *Escherichia coli* cultures carrying BAC clones was isolated using Qiagen Midi plasmid preparation kits (Qiagen). BAC restriction enzyme digestion products were resolved using CHEF gel electrophoresis (Chu et al. 1986; Copenhaver et al. 1995) using a CHEF-DR II pulsed-field gel electrophoresis system (Bio-Rad) and 1% certified megabase agarose (Bio-Rad) gels run in 50 mM TBE. Electrophoresis was performed at 200 V using a switching ramp time of 1–12 sec for 19 h in a 4°C cold room without additional cooling. After the run, the gel was stained with ethidium bromide (1.5 µg/mL in 50 mM TBE) for 40 min and imaged using a Gel Doc system (Bio-Rad).

### Bioinformatic analysis of Sf-2 rRNA gene sequences

DNA sequence reads of the Sf-2 genome were downloaded from the 19 Genomes Project Web site (http://mus.well.ox.ac.uk/19genomes) and mapped to the rRNA reference gene sequence (a Col-0 VAR1 gene) using Novaalign (version 3.00.03). Mapped reads were then assembled de novo into contiguous sequences using Newbler (version 2.7). Comparisons of Sf-2 contigs to the Col-0 reference sequence were conducted using NCBI BLAST (bl2seq), revealing ∼99% sequence identity.

## Supplementary Material

Supplemental Material
