# Supplementary material for: Chromosome-specific NOR inactivation explains selective rRNA gene silencing and dosage control in Arabidopsis
Source: Genes Dev. 2016 Jan 15;30(2):177–90. doi: 10.1101/gad.273755.115 (PMC4719308; doi:10.1101/gad.273755.115)
Supplement: Supplemental Material [file supp_30_2_177__index.html]

Chromosome-specific NOR inactivation explains selective rRNA gene silencing and dosage control in Arabidopsis — Chromosome-specific NOR inactivation explains selective rRNA gene silencing and dosage control in Arabidopsis — Supplemental Material 

# Chromosome-specific NOR inactivation explains selective rRNA gene silencing and dosage control in *Arabidopsis*

## Supplemental Material

**Files in this Data Supplement:**

- Supp Figure S1.pdf
- Supp Figure S11.pdf
- Supp Figure S2.docx
- Supp Figure S3.docx
- Supp Figure S4.ai
- Supp Figure S5.docx
- Supp Figure S6.ai
- Supp Figure S7.ai
- Supp Figure S8.ai
- Supp Figure S9.ai
- Supp Figure S10.ai
- Supp Fig Legends.docx
