# Supplementary material for: Chromosome-specific NOR inactivation explains selective rRNA gene silencing and dosage control in Arabidopsis
Source: Genes Dev. 2016 Jan 15;30(2):177–90. doi: 10.1101/gad.273755.115 (PMC4719308; doi:10.1101/gad.273755.115)
Supplement: Supplemental Material [file supp_gad.273755.115_Figure_S1_PCR_Primers.pdf]

Figure S1. Primers used in this study

Primers for CAPs assays to distinguish alleles of parental ecotypes in RIL populations

RIL Population	Marker name (see Fig 3)	Primers	Restriction enzyme
Col x Sha	c2_00069	ACCTTGACGACCAGTCTTGG Forward	*MlyI*
		CTCCGGATAAGTTGGAGTCG Reverse	
	c2_00078	CCTCCTTCTAGTTCTAGAGCAGGTC Forward	*MseI*
		CCGGTGAATCGAAATTCATGACTCGG Reverse	
	c4_00012	CTAAACATGCAGAACGGTAATTTACACGGG Forward	*ApoI*
		GGTATCAGTAAGACAGCGGAGAATGCAACCG Reverse	
	c4_00049	CTGTGAAGAATGCGCGTAAGAAGG Forward	*DraI*
		CCAAACATCCAACATTATATAGGGGC Reverse	
	c4_00086	GGGACCTCAATAGCTTCTTTTGGCTG Forward	*HinfI*
		GCTAACGTGTGTCTTCCCAATGG Reverse	
	c4_00090	CTTACTTTGACTCCGAGCAAGGAG Forward	*HinfI*
		AACTTTGCCCAAAAGGCAAAGGCC Reverse	
Col x Bur	c2_00074	TCGTGCAATCGTTCTGAAC Forward	*HinfI*
		CCCTTGCTGATTTTTGCATT Reverse	
	c4_00090	GCCTTTTGGGCAAAGTTATG Forward	*DraI*
		TGTTGCGAACAGAGGAAGATT Reverse	
	c4_00093	TTTCCCGCCTCTACATCAAC Forward	*MlyI*
		TTCATTTCGGCAATTCAAGA Reverse	
Col x Ler	c2_00069	ACCTTGACGACCAGTCTTGG Forward	*XbaI*
		CTCCGGATAAGTTGGAGTCG Reverse	
	c2_00078	CAGCCGTATTACCGGAGCTA Forward	*MseI*
		ACACCCGGTGAATCGAAAT Reverse	
	c4_00030	GGATAGTTTGGTCAACAAGAGAGGAGC Forward	*MspI*
		GGGAACAAGATTTCACTGGCTTGTTCAC Reverse	

Primers to amplify chromosomal markers to map theCol-0 / Sf-2 breakpoint in the introgression line

Primer name		Primers	
430	c4_00430	GCCGCTTAACCAGACTATGTTCTTGATTC Forward	*Sfa*NI
430	c4_00430	GGAAACAGTCTGAATTTTAGGGAACACTAAC Reverse	
580	c4_00580	AAACTTCTTCTTCTTCTCTCTCGCCATC Forward	*XmnI*
580	c4_00580	CCCGTGGGCGTGTTTCATCCATTAC Reverse	

Primers used for PCR and RT-PCR assays

Primer name	Primers
3'ETS variable region	GACAGACTTGTCCAAAACGCCCAC Forward
	CCTGGTCGAGGAATCCTGGACGATT Reverse
Actin	GAGAGATTCAGATGCCCAGAAGTC Forward
	TGGATTCCAGCAGCTTCCA Reverse
Subtype 3/4 - specific VAR	GACAGACTTGTCCAGAACGCCCACC Forward
	GAAGGAATCCTGGACGATTACTTGCTAGTTC Reverse

Primers for CAPS or dCAPS assays of gene subtype SNPs

Polymorphism	Primers	Restriction enzyme	RIL population used for mapping to NOR2 or NOR4
T2943A	GAGTCTGGGCAGTCCGTGGGGAACCCCCAAT Forward	*MluCI*	Col-0 x Bur-0
	GGACGGTCGGTCATTCCTCGTG Reverse		
G2982A	GAGTCTGGGCAGTCCGTGG Forward	*EcoRV*	Col-0 x Bur-0
	CAAGCAAGCCCATTCTCCTC Reverse		
T3439C	GGCATCTTTTCACCTCGTCGCCGAA Forward	*MspI*	Col-0 x Bur-0
	CCTAGGCGGGTGGAATCGATGCCCATGGGGACATCTCGGTATTTCGTGCCCG Reverse		
T4302C, C4456G, A4565T	GGCTGTCCCGAAGTATCTCGCG Forward	*AluI, NaeI, EcoRV*	Col-0 x Bur-0
	AAGGACGGATGAGCTTTGGCGGG Reverse		
A6645C	GATACCTGTCCAAAACAGAACGACCCGCG Forward	*SphI*	Col-0 x Sha
	ACTTCAGTTCGCAGCACAGCATCCG Reverse		
INDEL 6678+CAT	CGAGGGCACGTCTGCCTGGGTGTCACAAATCGTCGTCCCTCACGATT Forward	*HinfI*	Col-0 x Bur-0
	AAGTTCACCACCGCATGTCGGTACG Reverse		
G7122A	CGACTCTCGGCAACGGATA Forward	*MspI*	Col-0 x Bur-0
	CGAACGCCGAAGACGTCCGAT Reverse		
G307T	GACAGACTTGTCCAAAACGCCCAC Forward	*HindIII*	Col-0 x Sha
	CCTGGTCGAGGAATCCTGGACGATT Reverse		
