# Supplementary material for: Chromosome-specific NOR inactivation explains selective rRNA gene silencing and dosage control in Arabidopsis
Source: Genes Dev. 2016 Jan 15;30(2):177–90. doi: 10.1101/gad.273755.115 (PMC4719308; doi:10.1101/gad.273755.115)
Supplement: Supplemental Material [file supp_gad.273755.115_Figure_S11.pdf]

Figure S11: BAC clones and the rRNA gene variant types they contain

BAC clone	rRNA gene types	BAC clone	rRNA gene types	BAC clone	rRNA gene types	BAC clone	rRNA gene types
F1A16	2	F1H16	1	F2A17	2	F2J22	1,3
F1A18	3	F1H17	3	F2B1	1	F2J3	1,3
F1A19	1,3	F1H23	1,3	F2B14	2	F2J8	1
F1A20	1,3	F1I13	2	F2B19	2	F2J9	1
F1B14	1	F1I15	2	F2B5	2	F2K5	2
F1B23	1	F1I9	1,3	F2C3	1	F2L11	1,3
F1C17	1,3	F1J10	1	F2D7	2	F2L18	1,3
F1C20	1,3	F1J16	1,3	F2D8	2	F2L21	1,3
F1D2	3	F1K10	1	F2D9	1	F2M10	1
F1D24	1,3	F1K15	1,3	F2E13	1,3	F2M12	1,3
F1D8	1	F1K2	2	F2F3	2	F2M13	1
F1E1	1	F1L21	1,3	F2F7	3	F2M18	1,3
F1E12	1,3	F1M1	1	F2F8	1	F2N10	2
F1E13	3	F1M2	2	F2G13	1,3	F2N24	2
F1E20	1	F1M3	1,3	F2G18	1,3	F2N4	1
F1F11	1	F1N3	1	F2G3	1,3	F2O12	1,3
F1F16	1	F1N7	1	F2H8	3,4	F2O18	1,3
F1F17	1	F1O7	2	F2I20	1,3	F2O20	1
F1F21	2	F1O8	2	F2I24	2	F2O24	2
F1F4	1,3	F1P14	2	F2I6	1,3	F2O4	1
F1F5	1,3	F1P17	1	F2I8	1,3	F2P1	1,3
F1G11	1,3	F1P24	2	F2J17	1	F3A12	1,3
F1G17	1	F2A10	2	F2J21	1,3		
