# Supplementary material for: Chromosome-specific NOR inactivation explains selective rRNA gene silencing and dosage control in Arabidopsis
Source: Genes Dev. 2016 Jan 15;30(2):177–90. doi: 10.1101/gad.273755.115 (PMC4719308; doi:10.1101/gad.273755.115)
Supplement: Supplemental Material [file supp_gad.273755.115_Figure_S2_45S_ref_seq.docx]

GENESDEV/2015/273755; **Chandrasekhara et al.; Fig S2**

**Figure S2.** 45S rRNA gene reference sequence used in our study.

ccctcccctaaatcactccaaaaaaaacaatccccaattctacacaagtgtttctacactaacaaagcaacagctccttaacgaattcccaact ttacacgagctcgtctctcgaggttaaatgttattacttggtaagattccggacctcgccaagtgttttgaaaacccgcaacgctcgcaaaggtggatagtgagaataataagtgaagagacagacttgtccaaaacgcccaccacgaaggtgcatagtgagaagagtaagtcaagagatagacttgtccaaaaagaaacggaagagaaagcgtggggagacgctcacgaaggtgcatagtgagaagagtaagtcaagagacagacttgttcgaaaagaaacagaagagaatgcttggggttacactcacgaaggtgcatagtgagaagagtaagtcaagagacagacttgttcgaaaagaaacaaaagagaatgcttggggagatagaagtgtgagatagttctcaagctaagaaagttgtaaaagctaagaactagcatcaaatgatggatgaaacacaaggtagttgttgaaaagtcaaacacttggtgatatgaacacaaacgttcaatatgacaaacccatgccaagtaaagagaaaatgaaaactggtgattgttgcggaaatcgtccaggattcctcgaccaggacttgaaatcgtcgaggggaaaaaatcggttccgaggaatcgtcgatccggacttggaatcgtcgagaaaagtttaccgggtccgaggatttgtcgaccaggagtggaaatcgtcgagaaaaatctatcgggtccgaggaatcgtcgaccaggacgaggaatcgtcgaccgggtccgaggatttgtcgaccaggggttgaaatcgtcgaccaggtccgagacttcatcgaccgggtccgaggattcgtcgaccaggagtggaaatcgtcgagaaaaatctatcgggtccgaggaatcgtcgaccaggacgaggaatcgtcgaccgggtccgaggatttgtcgaccaggtccgagacttcatcgaccgggtccgaggattcgtcgaccaggtccgagacttcatcgaccgggtccaagtatttgattttattttggaaccggtgtctcctcagacatttcaatgtatgttggtgccaagagggaaaagggctataaagctatataggggtgggtgtgtgtagtgggaattttgcccgcacgcgcgcgcgcgaataagaataagaataagtaataagaataagaataagaaaaagaaaaaaaaaaaaaaaaaaaaaaaacaaaaactccatagagttgttttctcaatcgggtccgaggaatcgtcgatctggacttggaatcgtcgagaaaagtttaccgggtccgaaaatttgtcgaccaggagtggaaatcgtcgagaaaaatctatcgggtccgaggaatcgtcgaccaggacgaggaatcgtcgaccgggtccgaggatttgtcgaccaggggttgaaatagtcgaccaggtccgagacttcatcgaccgggtccgaggaatcgtcgaccaggagtggaaatcgtcgagaaaaatctatcgggtccgaggaatcgtcgaccaggacgaggaatcgtcgaccgggtccgaggatttgtcgaccaggtccgagacttcatcgaccgggtccgaggattcgtcgaccaggtccgagacttcatcgaccgggtccaagtatttgattttattttggaaccggtgtctcctcagacatttcaatgtatgttggtgccaagagggaaaagggctataaagctatataggggtgggtgtgtgtagtgggaattttgcccgcacgcgcgcgcgcgaataagaataagaataagtaataagaataagaataagaaaaagaaaaaaaaaaaaaaaaaaacaaaaacaaaaactccatagagttgttttctcaatcgggtccgaggaatcgtcgatctggacttggaatcgtcgagaaaagtttaccgggtccgaaaatttgtcgaccaggagtggaaatcgtcgagaaaaatctatcgggtccgaggaatcgtcgaccaggacgaggaatcgtcgaccgggtccgaggatttgtcgaccaggggttgaaatcgtcgaccaggtccgagacttcatcgaccgggtccgaggaatcgtcgaccaggacgatgaatggtcgatgaaaatctatcgggttcgaggaatggtcgaccaggggttgaaatcgtcgaccaggtccgagacttcatcgaccgtgtccgaggagtggtcgagggtttgtcgaccaggacgaggaatcgtcgaccgggtccgaggatttgtcgaccaggggttgaaatcgtcgaccaggaccgagaattcgtcgaccaggacggcggaaccctcgaccaggacgatgaatgggcgatgaaaatctatcgggttcgaggaatggtcgaccaggggttgaaatcctcgaccaggtccgagacttcatcgaccgggtccgaggattcgtcgaccaggacggccggatgtccgagaaaaaaaaatgttgccgaataactttcgaaaatcattggatatgatgcaatgttttgtgatcgaatctcttaaaatacatcaataaagagtttaggatgtcaagtttgcatcaaatatgcccacggagccccaactagaccatgaaaatccgatgttgtatcaggtcaaatgacctagctagaggtgtcaaaaaattatgaaaatttaccagaaaataggatttagtatccttatgatgcatgccaaaaagaattttcaaattccaagtatttcttttttcttggcaccggtgtctcctcagacatttcaatgtctgttggtgccaagagggaaaagggctattaagctatataggggggtgggtgttgagggagtctgggcagtccgtggggaaccccctttttcggttcggacttgggtagcgatcgagggatggtatcggatatcggcacgaggaatgaccgaccgtccggccgccgggattttcgccggaaaacttttccgggcacttttccggcgatcggttttgttgcctttttccgagttttctcagcagttctcggacaaaaactgctgaatcgtcgaggagaatgggcttgcttgcgtgggctgccattagttcttcgaggcgttagggtggcggcggtataaaagtgtcggagttttttcagcagttctcggacaaaaattgctgagtggccgagaagaatgggcgtgtcatgcgtgggctgacatggattcttcgaggcctaggggtggcggtatataacttgttcgcatgatattaccgagatgtccccacgggcatcttttcacctcgtcgccgaagagaatgggcgtgtcatggcatgggctgacatggattctcctaggccgtttgggtggcggtatagtcgtcttgcgcacgaaataccgagatgtccccatgggcatcgattccacccgcctaggttggatgggcgtgcttcgtcggaaagcatggatccgcctaggctgtcccgagtgtgagcgaggtgtgagtgtcgcccatgggcatcgacaccttgcggctaggaactggaacgagacgggtggcaaagatttcgagtagcacttcatactaccgtgggttttttaaaccttccgagttttgttgatgttattccgagaattagcaaaccgtaacgaagatgttcttggcaaccatcttttgatgggagtccggctgttcgatagccggccaagggtgatgaacgaaatgtgaacccttgtctcgcctaggttggatgggcgtgcttcgttggaaagcatggatccgcctaggctgtcccgagtgtgagcgaggtgtgagtgtcgcccatgggcatcgacaccttgcggctaggaactggaacgagacgggtagcaaagatttcgagtagcacttcatactaccgtgggttttttaaaccttcctagttttgttgatgttattccgagaattagcaaaccgtaacgaagatgttcttggcaaccatcttttgatgggagtccggctgttcgaaagccggccaagggtgatgaacgaaatgtgaacccttgtctcgcctaggttggatgggcgtgcttcgttggaaagcatggatccgcctaggctgtcccgaagtatctcgcgcttgtacggctttggctcggattcgtccgtcttctttcttcttagccgagtacttcggtagattagttggaacgattgatgattttgagttaattgaacgttcggcgtatgagtggtgatcggatagctagtgttcgtaggctccatgctcgcgcatcgaactacctaccacctatccttctcagttaattcacgggcgatgttacgctcgatgatgagttccggggcctgtgtttcgtacctaatttgaaggaattgttgagtttggtttacacctttgcccgcggcttctccttcgtggggaagtcgtgggctcaaacatcggcgcttgttcacctctcgtcatcgcatttgttgccttgctcgcattggtgaatgagttgcgggttgaaatctcggatgcggaaaagttgtcgacggtgactcgaagtgattcagtcccgccaaagctcatccgtccttcgggcaaaagatgacggtcaagacctcgtcctttctctctttccattgcgtttgagaggatgtggcggggaattgccgtgatcgatgaatgcTACCTGGTTGATCCTGCCAGTAGTCATATGCTTGTCTCAAAGATTAAGCCATGCATGTGTAAGTATGAACGAATTCAGACTGTGAAACTGCGAATGGCTCATTAAATCAGTTATAGTTTGTTTGATGGTAACTACTACTCGGATAACCGTAGTAATTCTAGAGCTAATACGTGCAACAAACCCCGACTTATGGAAGGGACGCATTTATTAGATAAAAGGTCGACGCGGGCTCTGGCTTGCTCTGATGATTCATGATAACTCGACGGATCGCATGGCCTCTGTGCTGGCGACGCATCATTCAAATTTCTGCCCTATCAACTTTCGATGGTAGGATAGTGGCCTACCATGGTGGTAACGGGTGACGGAGAATTAGGGTTCGATTCCGGAGAGGGAGCCTGAGAAACGGCTACCACATCCAAGGAAGGCAGCAGGCGCGCAAATTACCCAATCCTGACACGGGGAGGTAGTGACAATAAATAACAATACCGGGCTCTTTCGAGTCTGGTAATTGGAATGAGTACAATCTAAATCCCTTAACGAGGATCCATTGGAGGGCAAGTCTGGTGCCAGCAGCCGCGGTAATTCCAGCTCCAATAGCGTATATTTAAGTTGTTGCAGTTAAAAAGCTCGTAGTTGAACCTTGGGATGGGTCGGCCGGTCCGCCTTTGGTGTGCATTGGTCGGCTTGTCCCTTCGGTCGGCGATACGCTCCTGGTCTTAATTGGCCGGGTCGTGCCTCCGGCGCTGTTACTTTGAAGAAATTAGAGTGCTCAAAGCAAGCCTACGCTCTGGATACATTAGCATGGGATAACATCATAGGATTTCGATCCTATTGTGTTGGCTTCGGGATCGGAGTAATGATTAACAGGGACAGTCGGGGGCATTCGTATTTCATAGTCAGAGGTGAAATTCTTGGATTTATGAAAGACGAACAACTGCGAAAGCATTTGCCAAGGATGTTTTCATTAATCAAGAACGAAAGTTGGGGGCTCGAAGACGATCAGATACCGTCCTAGTCTCAACCATAAACGATGCCGACCAGGGATCAGCGGATGTTGCTTATAGGACTCCGCTGGCACCTTATGAGAAATCAAAGTTTTTGGGTTCCGGGGGGAGTATGGTCGCAAGGCTGAAACTTAAAGGAATTGACGGAAGGGCACCACCAGGAGTGGAGCCTGCGGCTTAATTTGACTCAACACGGGGAAACTTACCAGGTCCAGACATAGTAAGGATTGACAGACTGAGAGCTCTTTCTTGATTCTATGGGTGGTGGTGCATGGCCGTTCTTAGTTGGTGGAGCGATTTGTCTGGTTAATTCCGTTAACGAACGAGACCTCAGCCTGCTAACTAGCTACGTGGAGGCATCCCTTCACGGCCGGCTTCTTAGAGGGACTATGGCCGTTTAGGCCAAGGAAGTTTGAGGCAATAACAGGTCTGTGATGCCCTTAGATGTTCTGGGCCGCACGCGCGCTACACTGATGTATTCAACGAGTTCACACCTTGCCGACAGGCCCGGGTAATCTTTGAAATTTCATCGTGATGGGGATAGATCATTGCAATTGTTGGTCTTCAACGAGGAATTCCTAGTAAGCGCGAGTCATCAGCTCGCGTTGACTACGTCCCTGCCCTTTGTACACACCGCCCGTCGCTCCTACCGATTGAATGATCCGGTGAAGTGTTCGGATCGCGGCGACGTGGGTGGTTCGCCGCCCGCGACGTCGCGAGAAGTCCACTAAACCTTATCATTTAGAGGAAGGAGAAGTCGTAACAAGGTTTCCGTAGGTGAACCTGCGGAAGGATCATTGtcgatacctgtccaaaacagaacgacccgcgaaccaaagatcaccactctcggtgggccggtttcttagccgattccttgcccgccggatccgtggtttcgcgtatcggcatgatcgggagcttttatctcggtcttgtcgtgcgcgttgcttccggatatcacaaaaccccggcacgaaaagtgtcaaggaacatgcaaacgaacggctggcattcgcctccccggagacggagtgtgggcggatgctgtgctgcgaactgaagtctAAAACGACTCTCGGCAACGGATATCTCGGCTCTCGCATCGATGAAGAACGTAGCGAAATGCGATACTTGGTGTGAATTGCAGAATCCCGTGAACCATCGAGTCTTTGAACGCAAGTTGCGCCCCAAGCCTTCTGGCCGAGGGCACGTCTGCCTGGGTGTCACAAatcgtcgtccctcaccatcctttgctgatgcgggacggaagctggtctcccgtgtgttaccgcacgcgttggcctaaatccgagccaaggacgcctggagcgtaccgacatgcggtggtgaacttgatccattacattttatcggtcgctcttgtccggaagctgtagatgacccaaagtccatatagcgACCCCAGGTCAGGCGGGATTACCCGCTGAGTTTAAGCATATCAATAAGCGGAGGAAAAGAAACTAACAAGGATTCCCTTAGTAACGGCGAGCGAACCGGGAAGAGCCCAGCTTGAAAATCGGACGTCTTCGGCGTTCGAATTGTAGTCTGGAGAAGCGTCCTCAGCGACGGACCGGGCCTAAGTTCCCTGGAAAGGGGCGCCAGAGAGGGTGAGAGCCCGTCGTGCCCGGACCCTGTCGCACCACGAGGCGCTGTCTACGAGTCGGGTTGTTTGGGAATGCAGCCCCAATCGGGCGGTAAATTCCGTCCAAGGCTAAATACGGGCGAGAGACCGATAGCGAACAAGTACCGCGAGGTAAAGATGAAAAGGACTTTGAAAAGAGAGTCAAAGAGTGCTTGAAATTGTCGGGAGGGAAGCGGATGGGGGCCGGCGATGCGTCCTGGTCGGATGCGGAACGGAGCAATCCGGTCCGCCGATCGATTCGGGGCGTGGACCGACGCGGATTACGGTGGCGGCCTAAGCCCGGGCTTTTGATACGCTTGTGGAGACGTCGCTGCCGTGATCGTGGTCTGCAGCACGCGCCTAACGGCGTGCCTCGGCATCAGCGTGCTCCGGGCGTCGGCCTGTGGGCTCCCCATTCGACCCGTCTTGAAACACGGACCAAGGAGTCTGACATGTGTGCGAGTCAACGGGTGAGTAAACCCGTAAGGCGCAAGGAAGCTGATTGGCGGGATCCTCGCGGGTGCACCGCCGACCGACCTTGATCTTCTGAGAAGGGTTCGAGTGTGAGCATGCCTGTCGGGACCCGAAAGATGGTGAACTATGCCTGAGCGGGGTAAAGCCAGAGGAAACTCTGGTGGAAGCCCGCAGCGATACTGACGTGCAAATCGTTCGTCTGACTTGGGTATAGGGGCGAAAGACTAATCGAACCATCTAGTAGCTGGTTCCCTCCGAAGTTTCCCTCAGGATAGCTGGAGCTCGGACGCGAGTTCTATCGGGTAAAGCCAATGATTAGAGGCATTGGGGGCGCAACGCCTCGACCTATTCTCAAACTTTAAATAGGTAGGACGTGTCGGCTGCTTTGTTGAGCCGTCACACGGAATCGAGAGCTCCAAGTGGGCCATTTTTGGTAAGCAGAACTGGCGATGCGGGATGAACCGGAAGCCGGGTTACGGTGCCCAACTGCGCGCTAACCTAGAACCCACAAAGGGTGTTGGTCGATTAAGACAGCAGGACGGTGGTCATGGAAGTCGAAATCCGCTAAGGAGTGTGTAACAACTCACCTGCCGAATCAACTAGCCCCGAAAATGGATGGCGCTTAAGCGCGACCTATACCCGGCCGTCGGGGCAAGAGCCAGGCCTCGATGAGTAGGAGGGCGCGGCGGTCGCTGCAAAACCTAGGGCGCGAGGCGCGGAGCGGCCGTCGGTGCAGATCTTGGTGGTAGTAGCAAATATTCAAATGAGAACTTTGAAGGCCGAAGAGGGGAAAGGTTCCATGTGAACGGCACTTGCACATGGGTTAGTCGATCCTAAGAGTCGGGGGAAACCCGTCTGATAGCGCTTAAGCGAACTTCGAAAGGGGATCCGGTTAAAATTCCGGAACCGGGACGTGGCGGTTGACGGCAACGTTAGGGAGTCCGGAGACGTCGGCGGGGGCCTCGGGAAGAGTTATCTTTTCTGTTTAACAGCCTGCCCACCCTGGAAACGGCTCAGCCGGAGGTAGGGTCCAGCGGCTGGAAGAGCACCGCACGTCGCGTGGTGTCCGGTGCGCCCCCGGGCGCCCTTGAAAATCCGGAGGACCGAGTGCCGCTCACGCCCGGTCGTACTCATAACCGCATCAGGTCTCCAAGGTGAACAGCCTCTGGTCGATGGAACAATGTAGGCAAGGGAAGTCGGCAAAATGGATCCGTAACTTCGGGAAAAGGATTGGCTCTGAGGGCTGGGCTCGGGGGTCCCAGTTCCGAACCCGTCGGCTGTCAGCGGACTGCTCGAGCTGCTTCCGCGGCGAGAGCGGGTCGCCGGCTGCCGGCCGGGGGACGACTGGGAACGGCTCTCTCGGGAGCTTTCCCCGGGCGTCGAACAGTCAGCTCAGAACTGGTACGGACAAGGGGAATCCGACTGTTTAATTAAAACAAAGCATTGCGATGGTCCCTGCGGATGCTAACGCAATGTGATTTCTGCCCAGTGCTCTGAATGTCAAAGTGAAGAAATTCAACCAAGCGCGGGTAAACGGCGGGAGTAACTATGACTCTCTTAAGGTAGCCAAATGCCTCGTCATCTAATTAGTGACGCGCATGAATGGATTAACGAGATTCCCACTGTCCCTGTCTACTATCCAGCGAAACCACAGCCAAGGGAACGGGCTTGGCAGAATCAGCGGGGAAAGAAGACCCTGTTGAGCTTGACTCTAGTCCGACTTTGTGAAATGACTTGAGAGGTGTAGGATAAGTGGGAGCTTCGGCGCAAGTGAAATACCACTACTTTTAACGTTATTTTACTTACTCCGTGAATCGGAGGCCGGGGTACAACCCCTGTTTTTGGTCCCAAGGCTCGCTTCGGCGGGTCGATCCGGGCGGAGGACATTGTCAGGTGGGGAGTTTGGCTGGGGCGGCACATCTGTTAAAAGATAACGCAGGTGTCCTAAGATGAGCTCAACGAGAACAGAAATCTCGTGTGGAACAAAAGGGTAAAAGCTCGTTTGATTCTGATTTTCAGTACGAATACGAACCGTGAAAGCGTGGCCTATCGATCCTTTAGACTTCGGAATTTGAAGCTAGAGGTGTCAGAAAAGTTACCACAGGGATAACTGGCTTGTGGCAGCCAAGCGTTCATAGCGACGTTGCTTTTTGATCCTTCGATGTCGGCTCTTCCTATCATTGTGAAGCAGAATTCACCAAGTGTTGGATTGTTCACCCACCAATAGGGAACGTGAGCTGGGTTTAGACCGTCGTGAGACAGGTTAGTTTTACCCTACTGATGCCCGCGTCGCGATAGTAATTCAACCTAGTACGAGAGGAACCGTTGATTCGCACAATTGGTCATCGCGCTTGGTTGAAAAGCCAGTGGCGCGAAGCTACCGTGCGCTGGATTATGACTGAACGCCTCTAAGTCAGAATCCGGGCTAGAAGCGACGCATGCGCCCGCCGCCCGATTGCCGACCCTCAGTAGGAGCTTAGGCTCCAAAGGCACGTGTCGTTGGCTAAGTCCGTTCGGCGGAACGGTCGTTCGGACCGCCTTGAATTATAATTACCACCGAGCGGCGGGTAGAATCCTTTGCAGACGACTTAAATACGCGACGGGGTATTGTAAGTGGCAGAGTGGCCTTGCTGCCACGATCCACTGAGATTCAGCCCTTTGTCGCTAAGATTCGA
