# Supplementary material for: Chromosome-specific NOR inactivation explains selective rRNA gene silencing and dosage control in Arabidopsis
Source: Genes Dev. 2016 Jan 15;30(2):177–90. doi: 10.1101/gad.273755.115 (PMC4719308; doi:10.1101/gad.273755.115)
Supplement: Supplemental Material [file supp_gad.273755.115_Figure_S3.docx]

GENESDEV/2015/273755; Chandrasekhara et al, Figure S3

**Figure S3.** Frequency of cloned PCR products representing SNPs or the reference sequence at the indicated positions.

| **Sequence polymorphism** | **Frequency** |
| --- | --- |
| 2943T (reference) | 37/43 |
| **2943A** | 6/43 |
| 2982G (reference) | 39/43 |
| **2982A** | 4/43 |
| 3439T (reference) | 27/33 |
| **3439C** | 6/33 |
| 3511C (reference) | 27/45 |
| **3511T** | 18/45 |
| 4302T (reference) | 43/48 |
| **4302C** | 5/48 |
| 4456C (reference) | 45/48 |
| **4456G** | 3/48 |
| 4565A (reference) | 41/48 |
| **4565T** | 7/48 |
| 6645A (reference) | 25/29 |
| **6645C** | 4/29 |
| 6978 (reference) | 43/48 |
| **6978 (+CAT)** | 5/48 |
| 7122G (reference) | 42/46 |
| **7122A** | 4/46 |
| 307G (reference) | 74/127 |
| **307T** | 53/127 |
