# Supplementary figures and images for: Chromosome-specific NOR inactivation explains selective rRNA gene silencing and dosage control in Arabidopsis
Source: Genes Dev. 2016 Jan 15;30(2):177–90. doi: 10.1101/gad.273755.115 (PMC4719308; doi:10.1101/gad.273755.115)

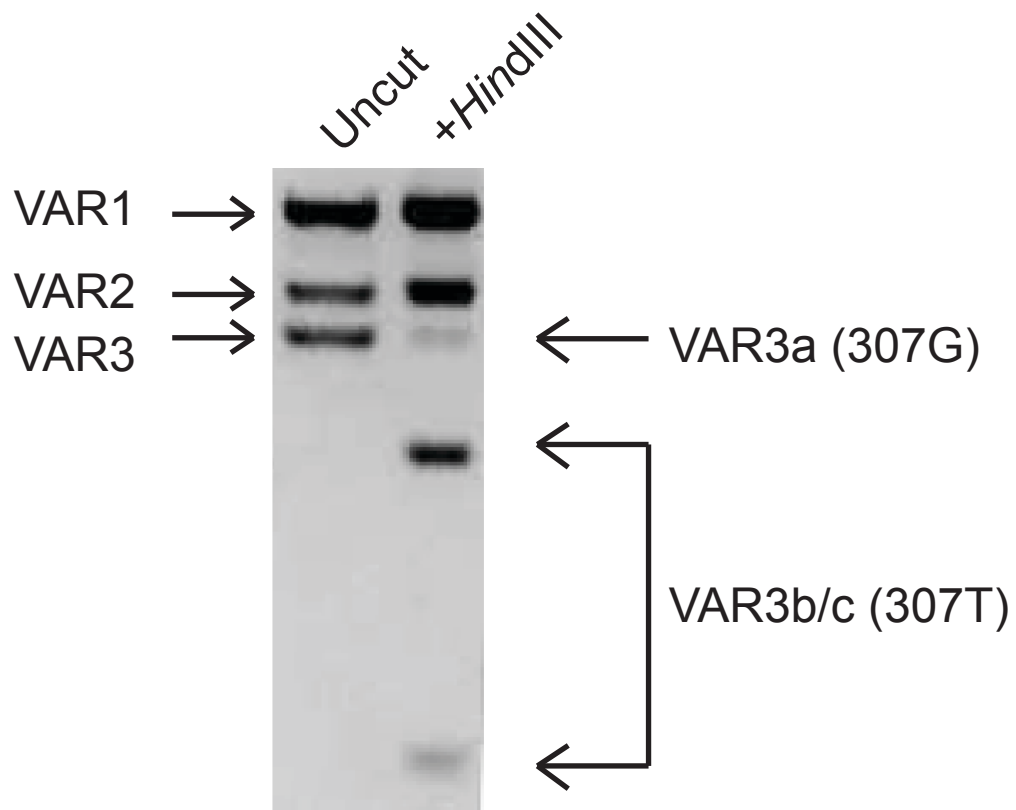

**Figure S4.** Estimation of SNP 307T occurrence in VAR1 and VAR3.

Supplement: Supplemental Material [file supp_gad.273755.115_Figure_S4.ps]

**Figure S6.** Segregation of VAR1 and VAR2 rRNA Genes in Col-0 x Sha RILs.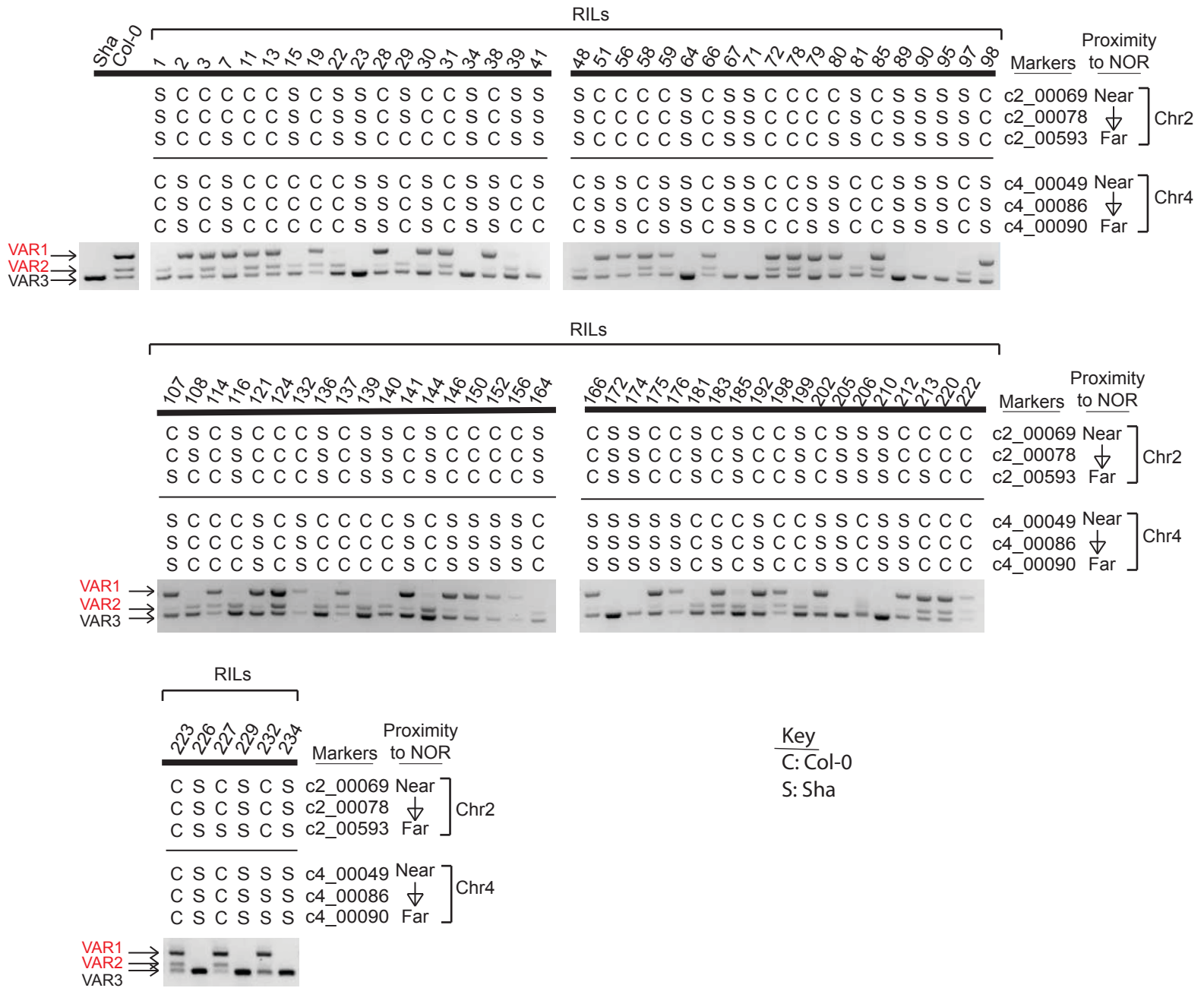

Supplement: Supplemental Material [file supp_gad.273755.115_Figure_S6_Col_x_Sha.ps]

Figure S7. Segregation of VAR2 and VAR3 rRNA Genes in Col-0 x Bur-0 Recombinant Inbred Lines

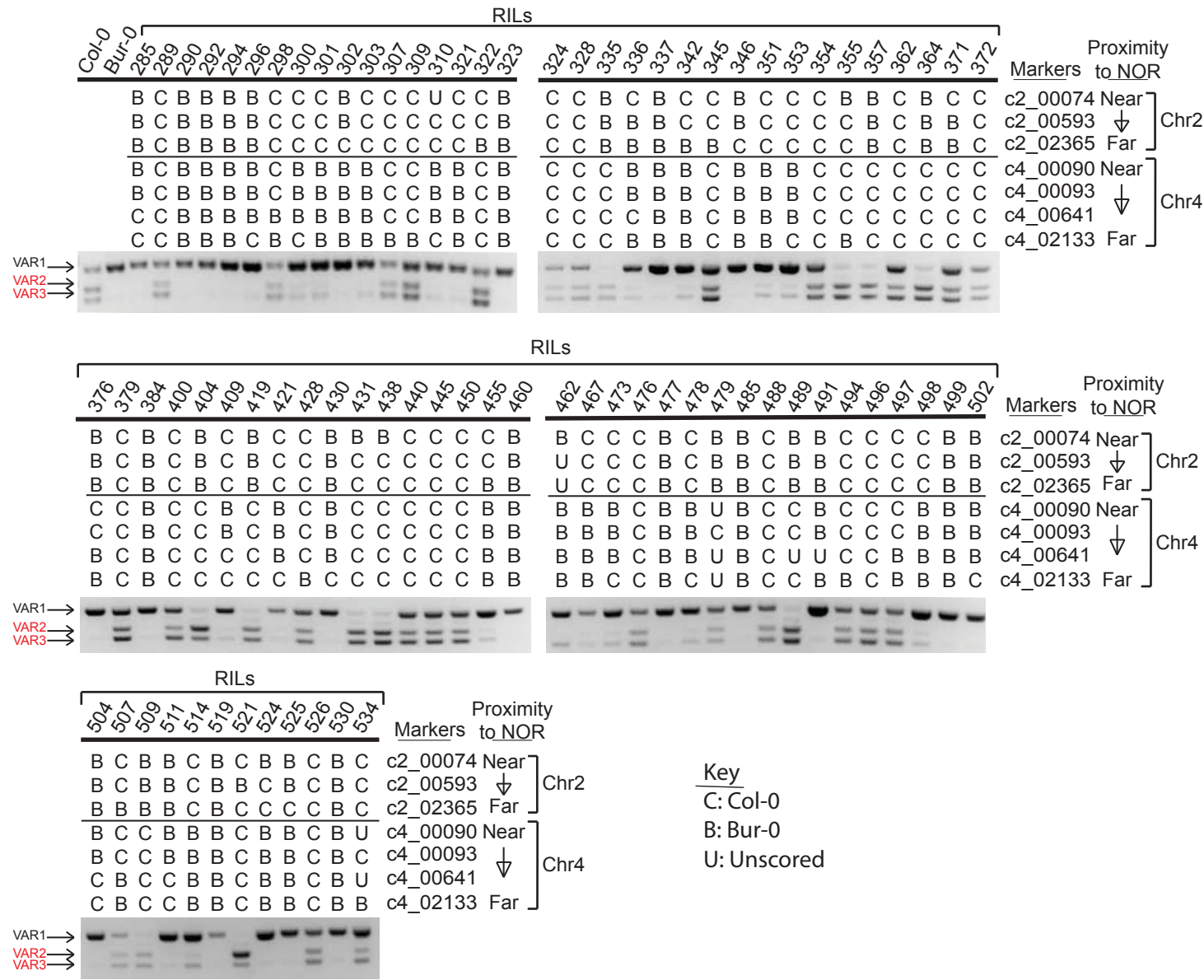

Supplement: Supplemental Material [file supp_gad.273755.115_Figure_S7_Col_x_Bur.ps]

Figure S9. Mapping rRNA genes bearing sequence polymorphisms in Col-0 x Bur-0 RILs.

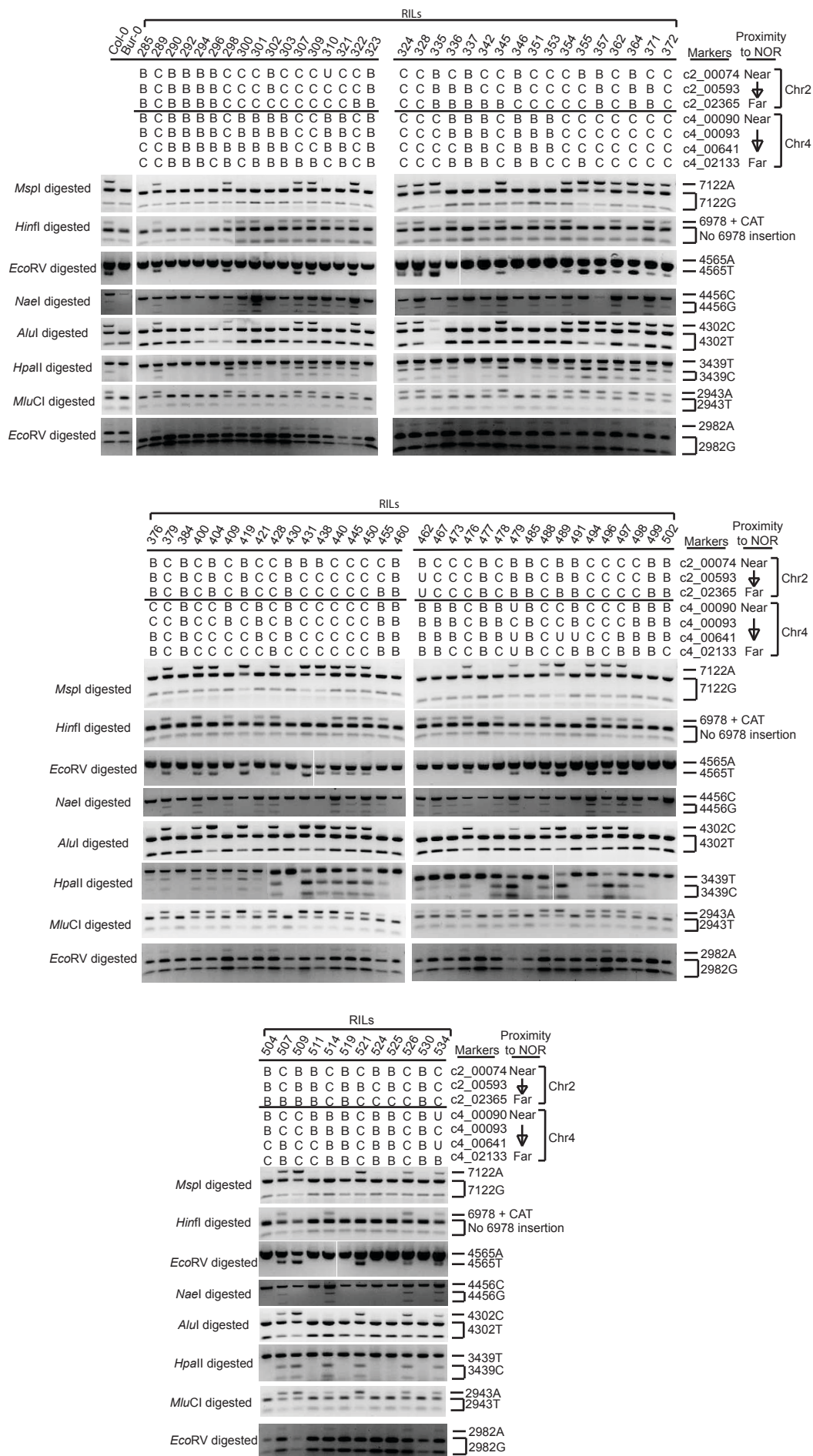

Supplement: Supplemental Material [file supp_gad.273755.115_Figure_S9_.ps]
