# Supplementary material for: Chromosome-specific NOR inactivation explains selective rRNA gene silencing and dosage control in Arabidopsis
Source: Genes Dev. 2016 Jan 15;30(2):177–90. doi: 10.1101/gad.273755.115 (PMC4719308; doi:10.1101/gad.273755.115)
Supplement: Supplemental Material [file supp_gad.273755.115_Figure_S5.docx]

GENESDEV/2015/273755; **Chandrasekhara et al, Figure S5**

**Figure S5.** Sequences of rRNA 3’ETS variable regions of *A. thaliana* ecotypes Col-0, Bur-0, Ler, and Sha.

**VAR1 class**

**Col-0 type 1**

GACAGACTTGTCCAAAACGCCCACCACGAAGGTGCATAGTGAGAAGAGTAAGTCAAGAGATAGACTTGTCCAAAAAGAAACGGAAGAGAAAGCGTGGGGAGACGCTCACGAAGGTGCATAGTGAGAAGAGTAAGTCAAGAGACAGACTTGTTCGAAAAGAAACAGAAGAGAATGCTTGGGGTTACACTCACGAAGGTGCATAGTGAGAAGAGTAAGTCAAGAGACAGACTTGTTCGAAAAGAAACAAAAGAGAATGCTTGGGGAGATAGAAGTGTGAGATAGTTCTCAAGCTAAGAAAGTTGTAAAAGCTAAGAACTAGCATCAAATGATGGATGAAACACAAGGTAGTTGTTGAAAAGTCAAACACTCGGTGATATGAACACAAACGTTCAATATGACAAACCCATGCCAAGTAAAGAGAAAATGAAAACTGGTGATTGTTGCGGAAATCGTCCAGGATTCCTCGACCAGG

**Col-0 type 2**

GACAGACTTGTCCAAAACGCCCACCACGAAGGTGCATAGTGAGAAGAGTAAGTCAAGAGATAGACTTGTCCAAAAAGAAACGGAAGAGAAAGCTTGGGGAGACGCTCACGAAGGTGCATAGTGAGAAGAGTAAGTCAAGAGATAGACTTGTTCAAAAAGAAACAGAAGAGAATGCTTGGGGTTACACTCACGAAGGTGCATAGTGAGAAGAGTAAGTCAAGAGACAGACTTGTTCAAAAAGAAACAAAAGAGAATGCTTGGGGAGATAGAAGTGTGAGATAGTTCTCAAGCTAAGAAAGTTGTAAAAGCTAAGAACTAGCATCAAATGATGGATGAAACACAAGGTAGTTGTTGAAAAGTCAAACACTTGGTGATATGAACACAAACGTTCAATATGACAAACCCATGCCAAGTAAAGAGAAAATGAAAACTGGTGATTGTTGCGGAAATCGTCCAGGATTCCTCGACCAGG

**Ler**

GACAGACTTGTCCAAAACGCCCACCACGAAGGTGCATAGTGAGAAGAGTAAGTCAAGAGATAGACTTGTCCAAAAAGAAACGGAAGAGAAAGCGTGGGGAGACGCTCACGAAGGTGCATAGTGAGAAGAGTAAGTCAAGAGACAGACTTGTTCGAAAAGAAACAGAAGAGAATGCTTGGGGTTACACTCACGAAGGTGCATAGTGAGAAGAGTAAGTCAAGAGACAGACTTGTTCGAAAAGAAACAAAAGAGAATGCTTGGGGAGATAGAAGTGTGAGATAGTTCTCAAGCTAAGAAAGTTGTAAAAGCTAAGAACTAGCATCAAATGATGGATGAAACACAAGGTAGTTGTTGAAAAGTCAAACACTTGGTGATATGAACACAAACGTTCAATATGACAAACCCATGCCAAGTAAAGAGAAAATGAAAACTGGTGATTGTTGCGGAAATCGTCCAGGATTCCTCGACCAGG

**Bur-0 type 1**

GACAGACTTGTCCAAAACGCCCACCACGAAGGTGCATAGTGAGAAGAGTAAGTCAAGAGATAGACTTGTCCAAAAAGAAACGGAAGAGAAAGCTTGGGGAGACGCTCACGAAGGTGCATAGTGAGAAGAGTAAGTCAAGAGATAGACTTGTTCAAAAAGAAACAGAAGAGAATGCTTGGGGTTACACTCACGAAGGTGCATAGTGAGAAGAGTAAGTCAAGAGACAGACTTGTTCGAAAAGAAACAAAAGAGAATGCTTGGGGAGATAGAAGTGTGAGATAGTTCTCAAGCTAAGAAAGTTGTAAAAGCTAAGAACTAGCATCAAATGATGGATGAAACACAAGGTAGTTGTTGAAAAGTCAAACACTTGGTGATATGAACACAAACGTTCAATATGACAAACCCATGCCAAGTAAAGAGAAAATGAAAACTGGTGATTGTTGCGGAAATCGTCCAGGATTCCTCGACCAGG

**Bur-0 type 2**

GACAGACTTGTCCAAAACGCCCACCGCGAAGGTGCATAGTGAGAAGAGTAAGTCAAGAGACAGACTTGTCCAAAAAGAAACGGAAGAGAAAGCTTGGGGAGACGCTCACGAAGGTGCATAGTGAGAAGAGTAAGTCAAGAGATAGACTTGTTCAAAAAGAAACAGAAGAGAATGCTTGGGGTTACACTCACGAAGGTGCATAGTGAGAAGAGTAAGTCAAGAGACAGACTTGTTCAAAAAGAAACAAAAGAGAATGCTTGGGGAGATAGAAGTGTGAGATAGTTCTCAAGCTAAGAAAGTTGTAAAAGCTAAGAACTAGCATCAAATGATGGATGAAACACAAGGTAGTTGTTGAAAAGTCAAACACTTGGTGATATGAACACAAACGTTCAATATGACAAACCCATGCCAAGTAAAGAGAAAATGAAAACTGGTGATTGTTGCGGAAATCGTCCAGGATTCCTCGACCAGG

**VAR4 class**

**Col-0**

GACAGACTTGTCCAAAACGCCCACCACGAAGGTGCATAGTGAGAAGAGTAAGTCAAGAGATAGACTTGTCCAAAAAGAAACGGAAGAGAAAGCTTGGGGAGACGCTCACGAAGGTGCATAGTGAGAAGAGTAAGTCAAGAGATAGACTTGTTCAAAAAGAAACAGAAGAGAATGCTTGGGGTTACACTCACGAAGGTGCATAGTGAGAAGAGTAAGTCAAGAGATAGACTTGTTCAAAAAGAAACAAAAGAGAATGCTTGGGGTTACACTCACGAAGGTGCATAGTGAGAAGAGTAAGTCAAGAGACAGACTTGTTCAAAAAGAAACAAAAGAGAATGCTTGGGGAGATAGAAGTGTGAGATAGTTCTCAAGCTAAGAAAGTTGTAAAAGCTAAGAACTAGCAAGTAATCGTCCAGGATTCCTCGACCAGG

**VAR2 class**

**Col-0**

GACAGACTTGTCCAAAACGCCCACCGCGAAGGTGCATAGTGAGAAGAGTAAGTCAAGAGATAGACTTGTTCAAAAAGAAACAGAAGAGAATGCTTGGGGTTACACTCACGAAGGTGCATAGTGAGAAGAGTAAGTCAAGAGACAGACTTGTTCAAAAAGAAACAAAAGAGAATGCTTGGGGAGATAGAAGTGTGAGATAGTTCTCAAGCTAAGAAAGTTGTAAAAGCTAAGAACTAGCATCAAATGATGGATGAAACACAAGGTAGTTGTTGAAAAGTCAAACACTTGGTGATATGAACACAAACGTTCAATATGACAACCCCATGCCAAGTAAAGAGAAAATGAAAACTGGTGATTGTTGCGGAAATCGTCCAGGATTCCTCGACCAGG

**VAR3 class**

**Col-0 type 1**

GACAGACTTGTCCAAAACGCCCACCACGAAGGTGCATAGTGAGAAGAGTAAGTCAAGAGATAGACTTGTCCAAAAAGAAACGGAAGAGAAAGCGTGGGGAGACGCTCACGAAGGTGCATAGTGAGAAGAGTAAGTCAAGAGACAGACTTGTTCGAAAAGAAACAGAAGAGAATGCTTGGGGTTACACTCACGAAGGTGCATAGTGAGAAGAGTAAGTCAAGAGACAGACTTGTTCGAAAAGAAACAAAAGAGAATGCTTGGGGAGATAGAAGTGTGAGATAGTTCTCAAGCTAAGAAAGTTGTAAAAGCTAAGAACTAGCAAGTAATCGTCCAGGATTCCTCGACCAGG

**Col-0 type 2**

GACAGACTTGTCCAAAACGCCCACCACGAAGGTGCATAGTGAGAAGAGTAAGTCAAGAGATAGACTTGTCCAAAAAGAAACGGAAGAGAAAGCTTGGGGAGACGCTCACGAAGGTGCATAGTGAGAAGAGTAAGTCAAGAGATAGACTTGTTCAAAAAGAAACAGAAGAGAATGCTTGGGGTTACACTCACGAAGGTGCATAGTGAGAAGAGTAAGTCAAGAGACAGACTTGTTCAAAAAGAAACAAAAGAGAATGCTTGGGGAGATAGAAGTGTGAGATAGTTCTCAAGCTAAGAAAGTTGTAAAAGCTAAGAACTAGCAAGTAATCGTCCAGGATTCCTCGACCAGG

**Ler**

GACAGACTTGTCCAAAACGCCCACCACGAAGGTGCATAGTGAGAAGAGTAAGTCAAGAGATAGACTTGTCCAAAAAGAAACGGAAGAGAAAGCTTGGGGAGACGCTCACGAAGGTGCATAGTGAGAAGAGTAAGTCAAGAGATAGACTTGTTCAAAAAGAAACAGAAGAGAATGCTTGGGGTTACACTCACGAAGGTGCATAGTGAGAAGAGTAAGTCAAGAGACAGACTTGTTCAAAAAGAAACAAAAGAGAATGCTTGGGGAGATAGAAGTGTGAGATAGTTCTCAAGCTAAGAAAGTTGTAAAAGCTAAGAACTAGCAAGTAATCGTCCAGGATTCCTCGACCAGG

**Sha**

GACAGACTTGTCCAAAACGCCCACCACGAAGGTGCATAGTGAGAAGAGTAAGTCAAGAGATAGACTTGTCCAAAAAGAAACGGAAGAGAAAGCTTGGGGAGACGCTCACGAAGGTGCATAGTGAGAAGAGTAAGTCAAGAGATAGACTTGTTCAAAAAGAAACAGAAGAGAATGCTTGGGGTTACACTCACGAAGGTGCATAGTGAGAAGAGTAAGTCAAGAGACAGACTTGTTCAAAAAGAAACAAAAGAGAATGCTTGGGGAGATAGAAGTGTGAGATAGTTCTCAAGCTAAGAAAGTTGTAAAAGCTAAGAACTAGCAAGTAATCGTCCAGGATTCCTCGACCAGG
