# Supplementary material for: Chromosome-specific NOR inactivation explains selective rRNA gene silencing and dosage control in Arabidopsis
Source: Genes Dev. 2016 Jan 15;30(2):177–90. doi: 10.1101/gad.273755.115 (PMC4719308; doi:10.1101/gad.273755.115)
Supplement: Supplemental Material [file supp_gad.273755.115_Figure_S8.ps]

Figure S8. Mapping the chromosomal position of VAR4 genes using a Col-0 x Sha F2 mapping population.

A. Specificity of a VAR3/VAR4-specific reverse primer (R2)

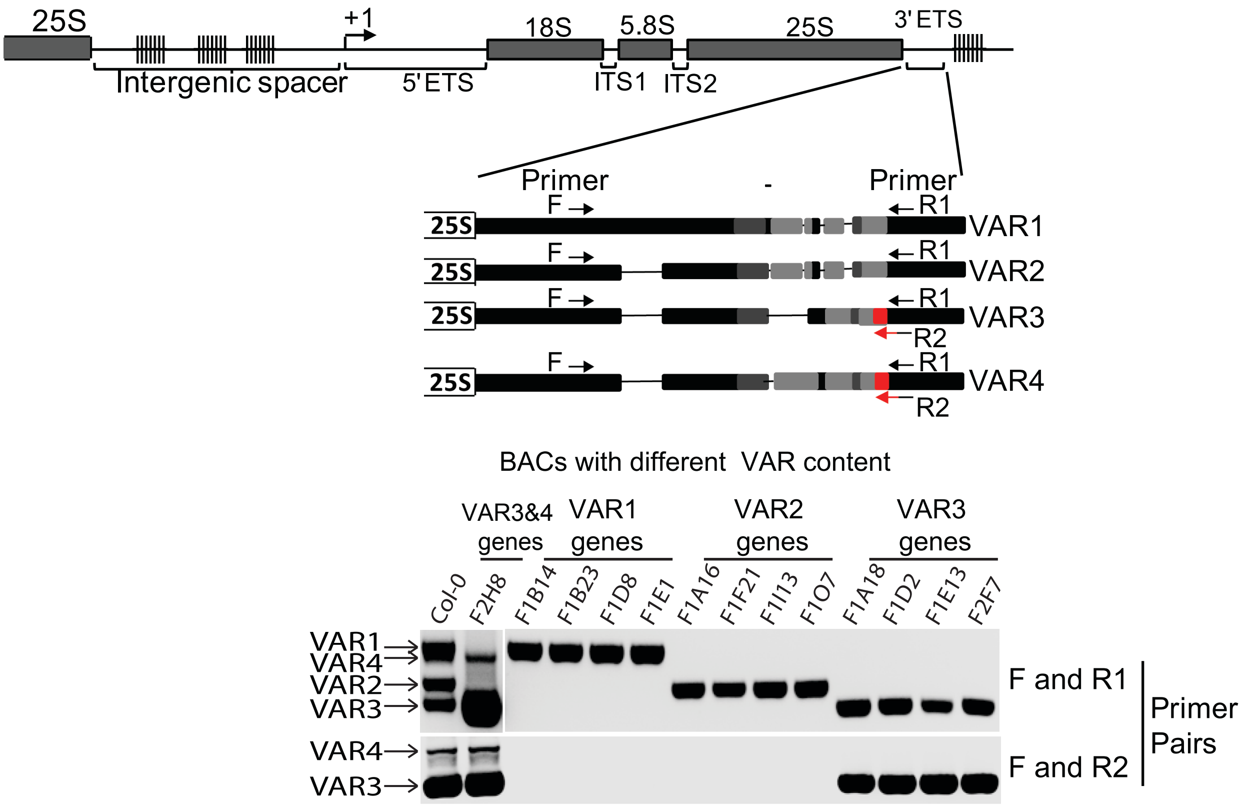

B. VAR4 mapping results in Col-0 x Sha F2 individuals with informative genotypes

	F2 individuals homozygous at each NOR				F2 individuals hetero-zygous at one NOR	
	Col-0 NOR2 Col-0 NOR4	Sha NOR2 Sha NOR4	Sha NOR2 Col-0 NOR4	Col-0 NOR2 Sha NOR4	Sha NOR2 Het NOR4	Het NOR2 Sha NOR4
# of individuals	5	5	2	8	16	13
# of individuals with VAR4 rRNA genes	5	0	2	0	16	0
