# Supplementary material for: Chromosome-specific NOR inactivation explains selective rRNA gene silencing and dosage control in Arabidopsis
Source: Genes Dev. 2016 Jan 15;30(2):177–90. doi: 10.1101/gad.273755.115 (PMC4719308; doi:10.1101/gad.273755.115)
Supplement: Supplemental Material [file supp_gad.273755.115_SuppFigLegends.docx]

**Supplemental Figure Legends**

**Figure S1.** PCR primers used in the study

**Figure S2.** 45S rRNA gene reference sequence used in our studies. The sequence represents a full-length rRNA gene repeat (VAR1 type) of *A.* *thaliana* ecotype Col-0. The sequence begins at the 5’ end of the intergenic spacer, just downstream of the 25S rRNA sequence and ends at the 3’ end of the 25S rRNA. Spacer sequences are shown in lower case; 18S, 5.8S and 25S rRNA sequences are shown in upper case letters.

**Figure S3.** Frequency of cloned PCR products representing SNPs or the reference sequence at the indicated positions. Independent PCR products that include the indicated polymorphic positions were cloned and sequenced. For each position, the less frequent polymorphism is shown in bold and the reference is shown in plain type. The fraction of clones containing the alternative polymorphisms is provided in the column at right.

**Figure S4.** Estimation of SNP 307T occurrence in VAR1 and VAR3.

Col-0 genomic DNA was subjected to PCR amplification using primers specific for the

3’ ETS variable region. *Hin*dIII-digested or undigested PCR products were then subjected

to agarose gel electrophoresis.

**Figure S5.** Sequences of rRNA 3’ETS variable regions of Col-0, Bur-0, Ler, and Sha. Genomic DNA was amplified by PCR and multiple independent clones were then sequenced. The frequencies of clones representing each of the sequences shown is provided in Figure 2C.

**Figure S6.** Segregation of VAR1 and VAR2 rRNA Genes in Col-0 x Sha Recombinant Inbred Lines. Genomic DNA was subjected to PCR amplification using primers flanking the 3’ ETS variable region. Products were resolved by agarose gel electrophoresis. Sha and Col-0 controls are shown at left in the top row.

**Figure S7. Segregation of VAR2 and VAR3 rRNA Genes in Col-0 x Bur-0 Recombinant Inbred Lines**

Genomic DNA was subjected to PCR amplification using primers flanking the 3’ ETS variable region. Products were resolved by agarose gel electrophoresis. Bur-0 and Col-0 controls are shown at left in the top row.

**Figure S8.** Mapping the chromosomal position of VAR4 genes using a Col-0 x Sha F2 mapping population.

A. Demonstration of the VAR3/VAR4 specificity of PCR amplification using reverse primer R2. The diagram shows the relative positions of the primers normally used to amplify the 3’ ETS variable region (F and R1) and of primer R2, whose 3’ end lies within an indel (shown in red) present only VAR3 and VAR4 genes. The gel images show PCR products obtained using primers F and R1 or F and R2 to amplify genomic DNA or DNA of BAC clones that contain VAR1 only, VAR2 only VAR3 only, or both VAR3 and VAR4.

B. Summary of VAR4 mapping results in Col x Sha F2 individuals with informative genotypes, demonstrating VAR4 association with Col-0 NOR4.

**Figure S9.** Mapping rRNA genes bearing sequence polymorphisms in Col-0 x Bur-0 RILs. Genomic DNA was amplified using primers flanking the polymorphic nucleotides indicated to the right of each row of images. DNA was then digested with a restriction endonuclease (indicated at the left of each row) that discriminates PCR products that differ with respect to the polymorphism. Col-0 and Bur-0 controls are shown at the left of each row in the top set of images.

**Figure S10.** Mapping VAR3a genes and genes bearing SNP 6645C in Col-0 x Sha recombinant inbred lines. Genomic DNA was amplified using primers flanking the 3’ETS variable region (for mapping VAR3a) or position 6645 C and resulting PCR products were then digested with *Hin*dIII or *Sph*I, respectively. Col-0 and Sha controls are shown at the left in the upper set of images.

**Figure S11.** List of BAC clones and the rRNA gene types they contain. Purified BAC DNA was subjected to PCR using the primer pair flanking the 3’ ETS variable region and resulting PCR products were subjected to agarose gel electrophoresis. The results are summarized in the table.
