# Supplementary material for: Chromosome-specific NOR inactivation explains selective rRNA gene silencing and dosage control in Arabidopsis
Source: Genes Dev. 2016 Jan 15;30(2):177–90. doi: 10.1101/gad.273755.115 (PMC4719308; doi:10.1101/gad.273755.115)

Figure S10. Mapping VAR3a genes and genes bearing SNP 6645C in Col-0 x Sha RILs

A. The VAR3a subtype of Col-0 is not cut by *Hind*III and is absent in ecotype Sha

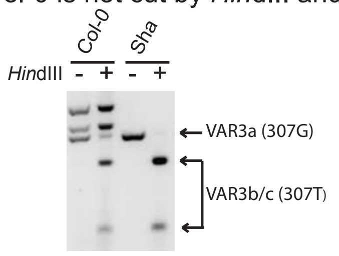

B. Mapping Col-0 Var3a, and SNP 6645C, in Col-0 x Sha recombinant inbred lines

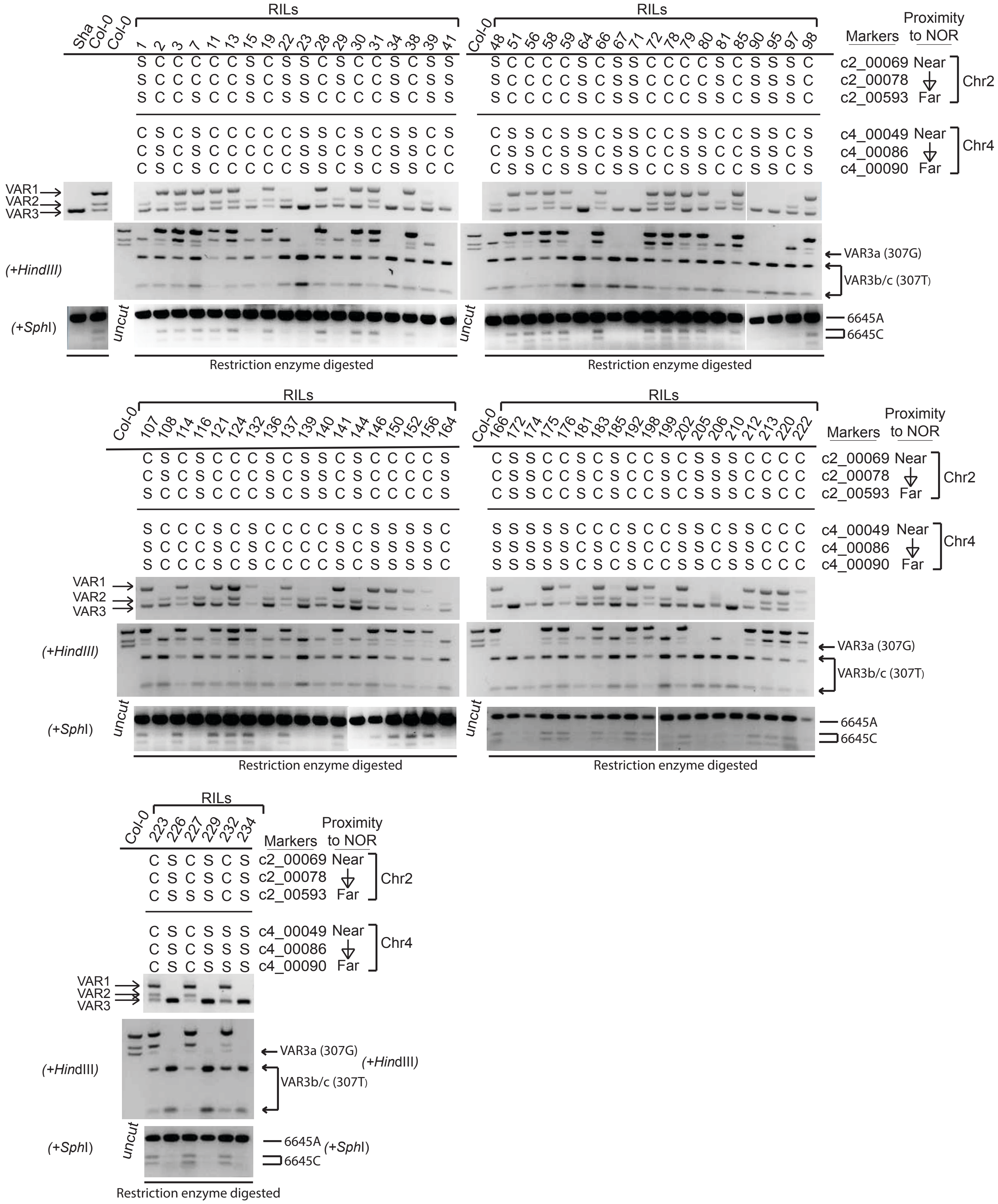

Supplement: Supplemental Material [file supp_gad.273755.115_Figure_S10.ps]
